# Triptoquinone A and B exercise a therapeutic effect in systemic lupus erythematosus by regulating NLRC3

**DOI:** 10.7717/peerj.15395

**Published:** 2023-06-09

**Authors:** Qinyao Xu, Xiangzhi Zhang, Shangqing Ge, Chang Xu, Yuanfan Lv, Zongwen Shuai

**Affiliations:** 1 Department of Rheumatology and Immunology, The First Affiliated Hospital of Anhui Medical University, Hefei, China; 2 Department of Laboratory, The First Affiliated Hospital of Anhui Medical University, Hefei, China; 3 Department of Internal Medicine, School Hospital of Anhui Medical University, Hefei, China

**Keywords:** Triptoquinone, Systemic lupus erythematosus (SLE), Oxidative stress, Inflammation, Apoptosis, NLRC3, Chondrocytes

## Abstract

The autoimmune disorder systemic lupus erythematosus (SLE) is multifaceted, with limited therapeutic alternatives and detrimental side effects, particularly on bones and joints. This research endeavors to examine the curative potential and underlying mechanisms of in addressing SLE-associated bone and joint complications. Triptoquinone A and triptoquinone B, constituents of *Tripterygium wilfordii* polyglycoside tablets (TGTs), exhibit antioxidant and anti-inflammatory attributes; nonetheless, its function in SLE therapy remains elusive. This investigation delves into the role of oxidative stress in systemic lupus erythematosus (SLE) and probes the prospective remedial effects of triptoquinone A and triptoquinone B on inflammation and cartilage deterioration in SLE-affected joints. Employing bioinformatics analyses, differentially expressed genes (DEGs) and protein-protein interactions were discerned in SLE, rheumatoid arthritis (RA), and osteoarthritis (OA) datasets. Enrichment analyses unveiled shared genes implicated in immune system regulation and toll-like receptor signaling pathways, among others. Subsequent examination of triptoquinone A and triptoquinone B revealed their capacity to diminish NLRC3 expression in chondrocytes, resulting in decreased pro-inflammatory cytokine levels and cartilage degradation enzyme expression. Suppression of NLRC3 augmented the protective effects of triptoquinone A and B, implying that targeting NLRC3 may constitute a potential therapeutic strategy for inflammation and cartilage degeneration-associated conditions in SLE patients. Our discoveries indicate that triptoquinone A and triptoquinone B may impede SLE progression via the NLRC3 axis, offering potential benefits for SLE-affected bone and joint health.

## Introduction

The autoimmune disorder systemic lupus erythematosus (SLE) is multifaceted, with limited therapeutic alternatives and detrimental side effects (Moulton et al., 2017; Ameer et al., 2022). SLE is associated with epigenetic and environmental factors (Wu, Chang & Lu, 2020). Articular involvement is a prevalent manifestation of SLE, ranging from 69% to 95% (Ceccarelli et al., 2017). This involvement gravely affects a patient’s quality of life, resulting in work disability and hindered daily functioning, imposing a significant burden on both individuals and society (Abu Bakar et al., 2019; Baker & Pope, 2008). Glucocorticoids, immunosuppressive agents, and biologics are common treatments for autoimmune diseases like rheumatoid arthritis (RA) and SLE (Ostrov, 2015). For instance, prednisone inhibits connective tissue proliferation, diminishes inflammatory exudation, and attenuates the inflammatory response; however, it can produce adverse effects and prove ineffective. By examining similarities to RA, we sought to assess potential pathogenic impacts and identify more specific biological pathways in disease development (van der Woude & van der Helm-van Mil, 2018).

Current research reveals that herbal extracts represent a promising avenue for treating systemic diseases (Zhang et al., 2019a; Jabbari et al., 2020; Tang, Hosein & Mattioli-Belmonte, 2021; Pandey & Nimisha, 2020). Traditional medicine (TCM) has demonstrated superior efficacy in addressing locomotor system-related diseases and autoimmune disorders in recent years (Jabbari et al., 2020; Pandey & Nimisha, 2020; Zhou et al., 2010; Li et al., 2021; Chen et al., 2022a). *Tripterygium wilfordii* Hook F is renowned for its dehumidiferous roots, which produce Tripterygium glycosides (TGTs). Contemporary pharmacological studies have revealed that TGTs also possess specific anti-inflammatory and immunomodulatory properties (Wang et al., 2017).

Triptoquinone A and triptoquinone B are constituent components of TGTs. Triptoquinone-A, derived from *Tripterygium wilfordii*, has been shown to inhibit nitric oxide synthase induction by endotoxin and interleukin-1 beta, effectively preventing arginine-induced vasorelaxation in vascular smooth muscle (Moritoki et al., 1996). Triptoquinone exhibits a multitude of effects, including antioxidant and anti-inflammatory properties (Ziaei & Halaby, 2016; Sierra et al., 2022). However, investigations examining the specific mechanisms of triptoquinone in SLE treatment remain scarce. This study delved into the therapeutic impact of triptoquinone and unveiled the association between the inflammatory response and apoptosis, as well as oxidative stress in SLE treatment through cell and animal experiments. The mode of action of triptoquinone A and triptoquinone B as biomaterials in SLE-associated arthritis, nonetheless, is still enigmatic, and further clarification of their active constituents is warranted.

Bioinformatics approaches and cell experiments methods hold vital roles and significance in identifying target genes for diseases and screening targeted drugs (Li et al., 2019; Tao et al., 2022; Lu et al., 2022; Kang et al., 2022; Huang et al., 2022; Chen et al., 2022b). This study aims to employ bioinformatics and cell experiment to identify potential therapeutic targets for preventing the development of SLE-related arthritis.

## Materials & Methods

### Bioinformatics analysis

The GEO database was utilized to identify co-expression genes from SLE, rheumatoid arthritis (RA), and osteoarthritis (OA). The screening criteria included: (i) “SLE”, “RA”, and “OA”; (ii) human; and the data were incorporated into the study. Ultimately, the study included 30 patients diagnosed with SLE and 25 individuals who were not afflicted with SLE or RA, as determined from the GSE81622 microarray dataset (Zhu et al., 2016). Additionally, 16 patients and 7 unaffected individuals from the GSE77298 microarray dataset, as well as 18 patients and 20 unaffected individuals from the GSE114007 microarray dataset with OA, were incorporated into this investigation (Broeren et al., 2016; Fisch et al., 2018). A *P*-value of less than 0.05 and a —log2 FC— greater than or equal to 1.00 were used to identify differentially expressed genes (DEGs). Limma R package identified DEGs between SLE and normal samples. The ClusterProfiler R package assessed Gene Ontology (GO) terms and Kyoto Encyclopedia of Genes and Genomes (KEGG) pathways for DEGs. A total of 1,399 genes associated with oxidative stress were gathered from GeneCard (http://www.genecards.org).

### Screening of co-expressed DEGs and construction of PPI network

R software and Perl software program were applied to intersect “SLE”, “RA”, and “OA” related differentially expressed genes for intersection analysis and input into Venny 2.1 software (http://bioinfogp.cnb.csic.es/tools/venny/index.html) to create a Venn diagram. In order to construct Protein-Protein Interaction (PPI) of co-expressed genes, we used the STRING database (https://string-db.org/). In order to screen key genes in the PPI network, Cytoscape 3.7.2 software (https://cytoscape.org/) was used.

### Culturing and identification of human chondrocytes

Our human chondrocytes were obtained from the Shanghai-based Chinese Academy of Sciences’ Institute of Cell Research. The murine chondrogenic cell line, designated as ATDC5 cells, was procured from the reputable Nanjing Cobioer Biotechnology Co., Ltd. All chondrocytes were meticulously suspended in Dulbecco’s Modified Eagle Medium, which encompassed a 15% admixture of fetal bovine serum, sourced from Clark Bioscience, USA, as well as a Penicillin-Streptomycin-Amphotericin B Solution, originating from Beyotime (Beijing, China). Cell cultivation transpired within a humidified incubator, maintained at 37 °C and supplemented with 5% CO2. Human chondrocyte cells were subjected to a pre-treatment involving lipopolysaccharide at a concentration of 100 ng/mL, with a duration of 8 h. Concurrently, ATDC5 cells were induced utilizing 10 ng/ml of IL-1 *β* for 24 h, thereby establishing an osteoarthritis model that was sustained *in vitro*. All cells were then treated with triptoquinone A or triptoquinone B at concentrations ranging from 10 mM to 30 mM. Following an 8-hour treatment period, cell lines underwent comprehensive RNA and protein extraction, thereby enabling the progression of subsequent experimental procedures. Untreated cells and cells treated with DMSO served as controls.

### Vitality of the cell

Triptoquinone was assessed on chondrocyte viability using the Cell-Counting Kit-8 (CCK-8; Dojindo, Kumamoto, Japan). Initially, human chondrocytes (5,000 cells/well) were incubated for 24 h after inoculation into 96-well plates. Phosphate-buffered saline (PBS; Biosharp, Hefei City, China) was used to wash all chondrocytes after discarding the medium. The chondrocytes were then incubated with triptoquinone for 24 h. Lastly, 10 µL of The enzyme marker was used to measure absorption at 450 nm after adding CCK-8 to each well and incubating for two hours.

### Western Blot (WB)

At 4 °C, cells treated with triptoquinone A and triptoquinone B were washed with PBS, and RIPA+ protease inhibitor was added. After thorough mixing for 30 min, corresponding protein suspensions were obtained, and supernatants were collected after centrifugation. The obtained supernatant was combined with loading buffer and further treated at 100 °C for 10 min before loading. Equal amounts of proteins were electrophoresed using a 10% SDS-PAGE gel, transferred onto PVDF, and the transferred PVDF membrane was blocked with a 5% skim milk powder emulsion for 2 h. Incubation of primary antibodies at 4 °C was performed overnight. The primary antibodies used included anti-NLRC3 (1:1000, CST, China) and anti-GAPDH (1:5000, CST). Following incubation, images were taken and stored using a developer (Tanon, Shanghai, China), after adding secondary antibodies and incubating them at room temperature for two hours.

### Extraction of total cellular RNA and qRT-PCR analysis

Total RNA was extracted using Trizol (Beyotime, Jiangsu, China) from cells treated with triptoquinone A and triptoquinone B. The cells were washed in PBS after being treated with triptoquinone A and triptoquinone B, and RIPA+ protease inhibitor was added at 4 °C. The cDNA was subsequently extracted using the TB Green Premix Ex. We used GAPDH as an internal reference to calculate the relative expression of mRNA.

### NLRC3-siRNA transfection

NLRC3 gene silencing was achieved through the utilization of NLRC3-siRNA (Santa Cruz Biotechnology, Dallas, TX, USA) (Kang et al., 2020). According to the manufacturer’s instructions, chondrocytes were seeded into 6-well plates and incubated for 24 h. Subsequently, transfection with negative control and NLRC3 double-stranded siRNA was performed using Lipofectamine 2000 siRNA transfection reagent (Thermo Fisher, Waltham, MA, USA) at a concentration of 50 nM for 36 h, respectively. The effectiveness of the transfection process was verified through RT-PCR and WB analysis.

### Cell transfection

Cell transfection was conducted employing Liposome 3000 reagent (Invitrogen, Waltham, MA, USA), in accordance with the manufacturer’s directives. For NLRC3 downregulation, and their corresponding negative controls, materials were obtained from Sangon Biotech. Cells underwent transfection for 24 h.

### Intracellular ROS assessment

A 24-well plate of ATDC5 cells was incubated for 2 h in IL-1b (10 ng/mL) for 24 h to examine intracellular ROS accumulation. After discarding the medium, the cells were rinsed and incubated with DCF-DA for 30 min. Following cell washing, intracellular ROS activity was evaluated using a Synergy HT Microplate Reader.

### Malondialdehyde (MDA) evaluation

ATDC5 cells were cultured in 24-well plates for 24 h in IL-1b (10 ng/mL) and then rinsed and homogenized after treatment. Protein content of the supernatant was determined using the Bradford method after centrifugation of the homogenate. In total, 100 mL of supernatant was amalgamated with 1.5 mL acetic acid (20%), 1.5 mL thiobarbituric acid (0.8%), and 200 mL sodium dodecyl sulfate (8%) in 100 ml. The reaction mixtures were heated for 60 min at 95 °C, cooled to room temperature, and then n-butanol was added to each. Following mixing and centrifugation at 3,000 g for 10 min, the organic layer was retrieved, and absorbance was measured at 532 nm.

### Statistical analysis

All experiments in this investigation were independently executed three times, with data subjected to analysis using SPSS 26.0 statistical software. Data were displayed as mean ± standard deviation. A *p*-value <0.05 was deemed statistically significant. Statistical analysis was performed with the Student’s *t*-test for comparisons between two groups, and a one-way ANOVA for comparisons among multiple groups.

## Results

### Bioinformatics analysis of disease-associated genes.

**Figure 1:**
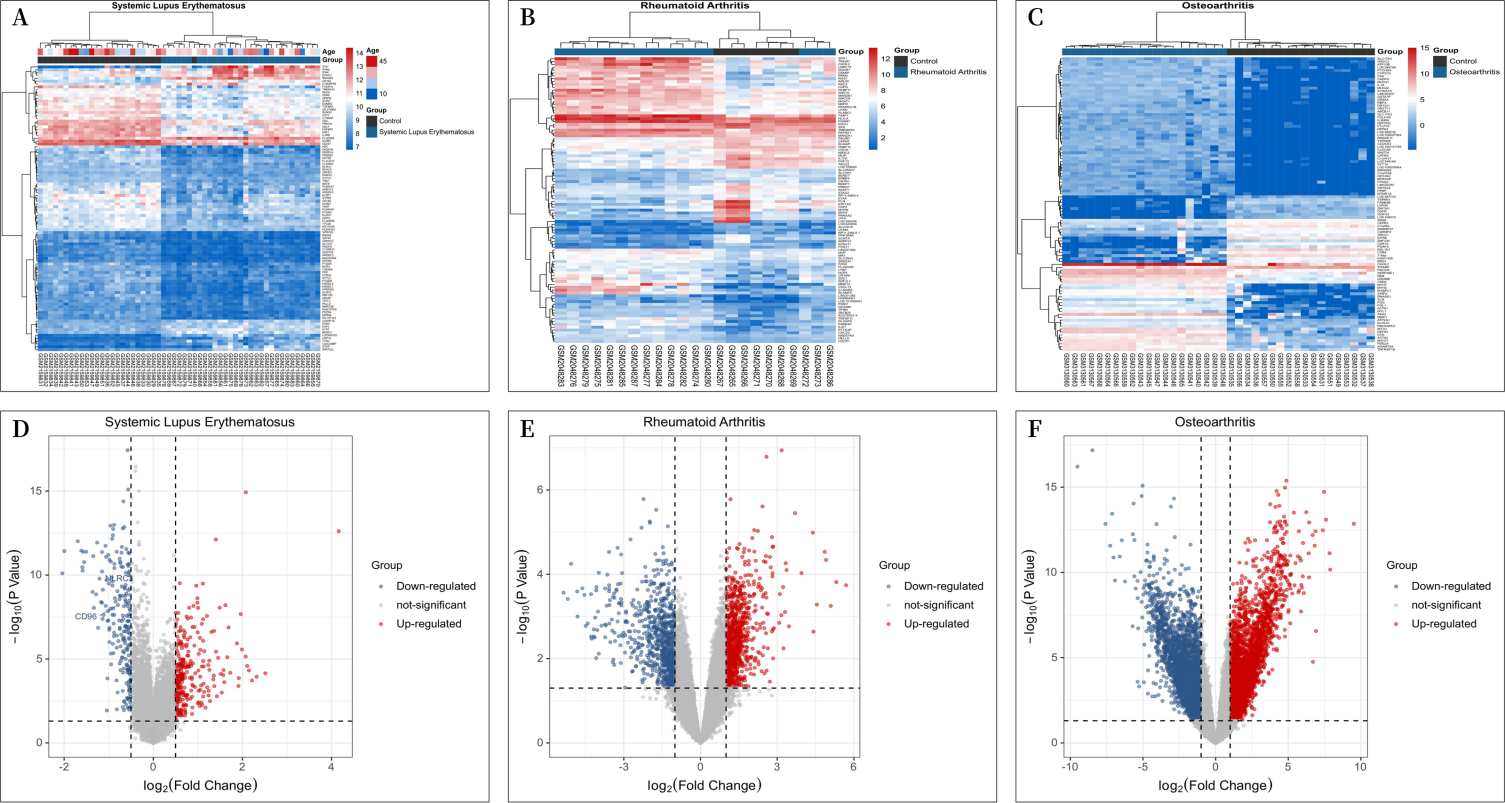
Differential Expression Heat Maps and Volcano Plots. (A) Heat map of differentially expressed genes (DEGs) in GSE81622; (B) Heat map of DEGs in GSE77298; (C) Heat map of DEGs in GSE114007; (D) Volcano plot of DEGs in GSE81622; (E) Volcano plot of DEGs in GSE77298; (F) Volcano plot of DEGs in GSE114007.

A total of 30 patients with “SLE” and 25 “individuals without SLE or RA” from the GSE81622 microarray dataset, 16 patients with RA and “individuals without SLE or RA” from the GSE77298 microarray dataset, 16 patients and 7 “individuals without SLE or RA” from the GSE77298 microarray dataset, and 18 patients and 20 “individuals without SLE or RA” from the GSE114007 microarray dataset with OA were included in the study. The criterion for screening DEGs was a *P* value <0.05 and —log2 fold change (FC)—≥1.00. GSE81622 screened 345 differentially expressed mRNAs, including 203 upregulated and 142 downregulated genes. GSE77298 included 1,717 differentially expressed mRNAs, including 891 upregulated mRNAs and 826 downregulated mRNAs, and GSE114007 included 1646 differentially expressed mRNAs. According to the *P* value, the 100 most significantly differentially expressed mRNAs in Figs. 1A–1C are displayed. And the processed data were imported into R to plot volcano plots (Figs. 1D–1F).

### Shared DEGs screening and PPI network construction

**Figure 2:**
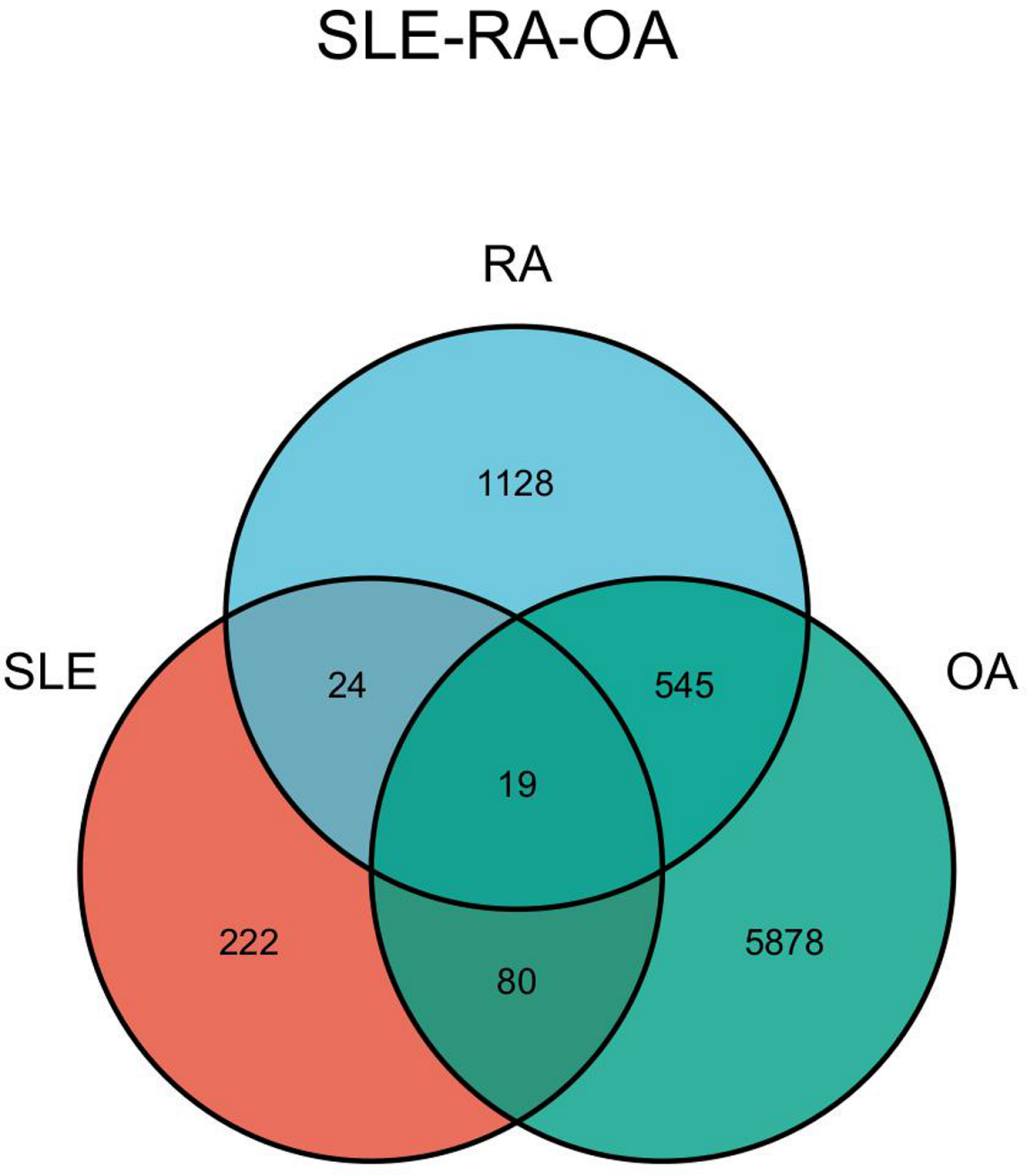
Venn diagram illustrating the co-genes associated with SLE, RA, and OA.

**Figure 3:**
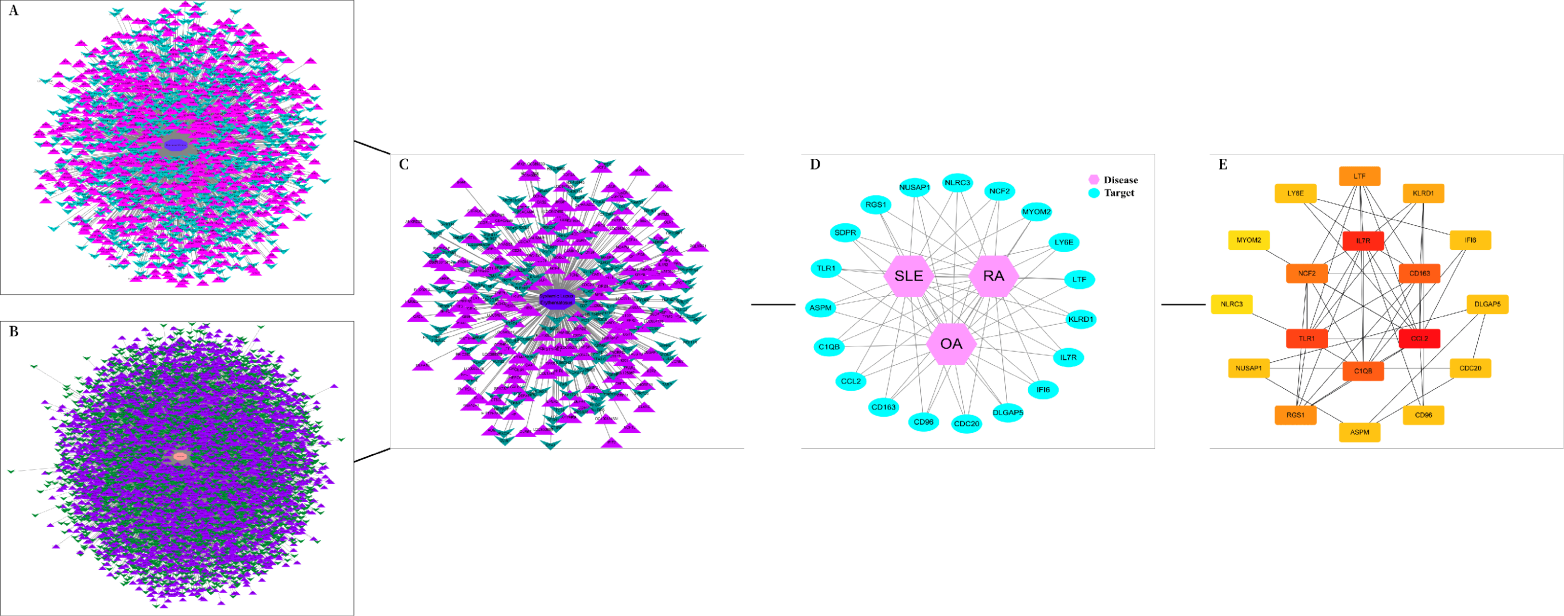
DEGs interaction networks. (A) Interaction network of DEGs in GSE77298; (B) Interaction network of DEGs in GSE114007; (C) Interaction network of DEGs in GSE81622; (D) PPI network of disease co-expressed genes; (E) Top 18 potential core DEGs calculated based on degree values.

The DEGs of the SLE-related GSE81622 dataset, RA-related GSE77298 dataset, and OA-related GSE114007 dataset were imported into the online Venn diagram production website Venny, resulting in 19 common DEGs between “SLE”, RA, and OA (Fig. 2). Protein-protein interaction (PPI) networks of DEGs related to “SLE”, RA, and OA were independently constructed using Cytoscape 3.7.2 software. STRING was used to combine DEGs from the three datasets, and unconnected targets were hidden under settings with “medium confidence = 0.400” selected. The PPI result data was then exported (Figs. 3A–3D) (Chin et al., 2014).

### GO and KEGG enrichment analysis

**Figure 4:**
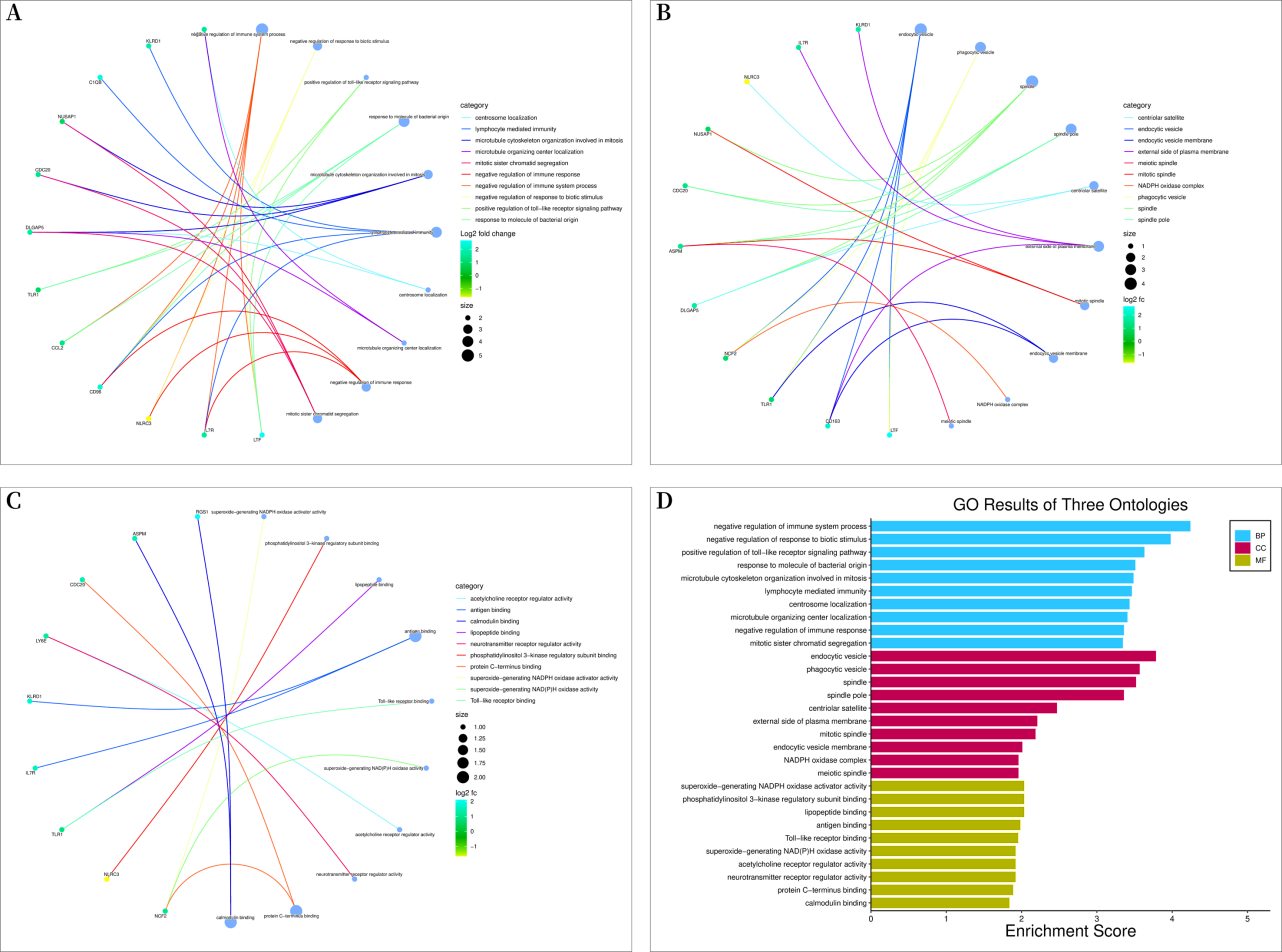
Gene ontology (GO) enrichment analysis. (A) Chord diagram of biological processes (BP); (B) chord diagram of cellular components (CC); (C) chord plot of molecular functions (MF); (D) histogram of GO enrichment analysis.

**Figure 5:**
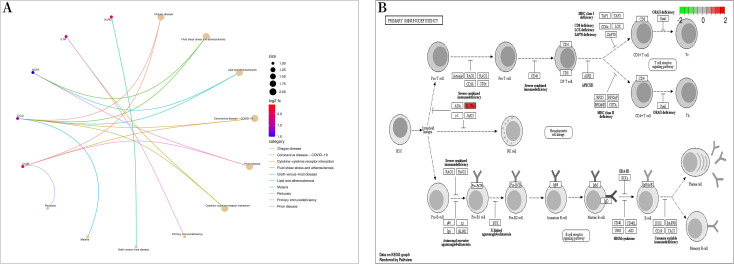
Kyoto encyclopedia of genes and genomes (KEGG) enrichment analysis. (A) Bubble diagram of KEGG enrichment analysis; (B) primary immunodeficiency signaling pathway.

Bioconductor and clusterProfiler packages of R software were used to analyze the enrichment of GO and KEGG pathways between “SLE”, RA, and OA. The GO analysis indicated that their biological processes (BP) were primarily enriched in negative regulation of immune system processes, negative regulation of response to biotic stimuli, positive regulation of toll-like receptor signaling pathways, among others (Fig. 4A). Cellular components (CC) were mainly enriched in endocytic vesicles, phagocytic vesicles, and spindles (Fig. 4B); molecular functions (MF) were primarily enriched in superoxide-generating NADPH oxidase activator activity, phosphatidylinositol 3-kinase regulatory subunit binding, and lipopeptide binding (Figs. 4C–4D). KEGG pathway enrichment analysis identified 19 shared genes, mainly focusing on fluid shear stress and atherosclerosis, lipid and atherosclerosis, coronavirus disease (COVID-19), cytokine-cytokine receptor interactions, primary immunodeficiency, and other immune-related signaling pathways (Fig. 5A). The pathview package was used to display the signaling pathways related to “SLE”, RA, and OA (Fig. 5B).

### Triptoquinone A and triptoquinone B attenuate NLRC3 expression

**Figure 6:**
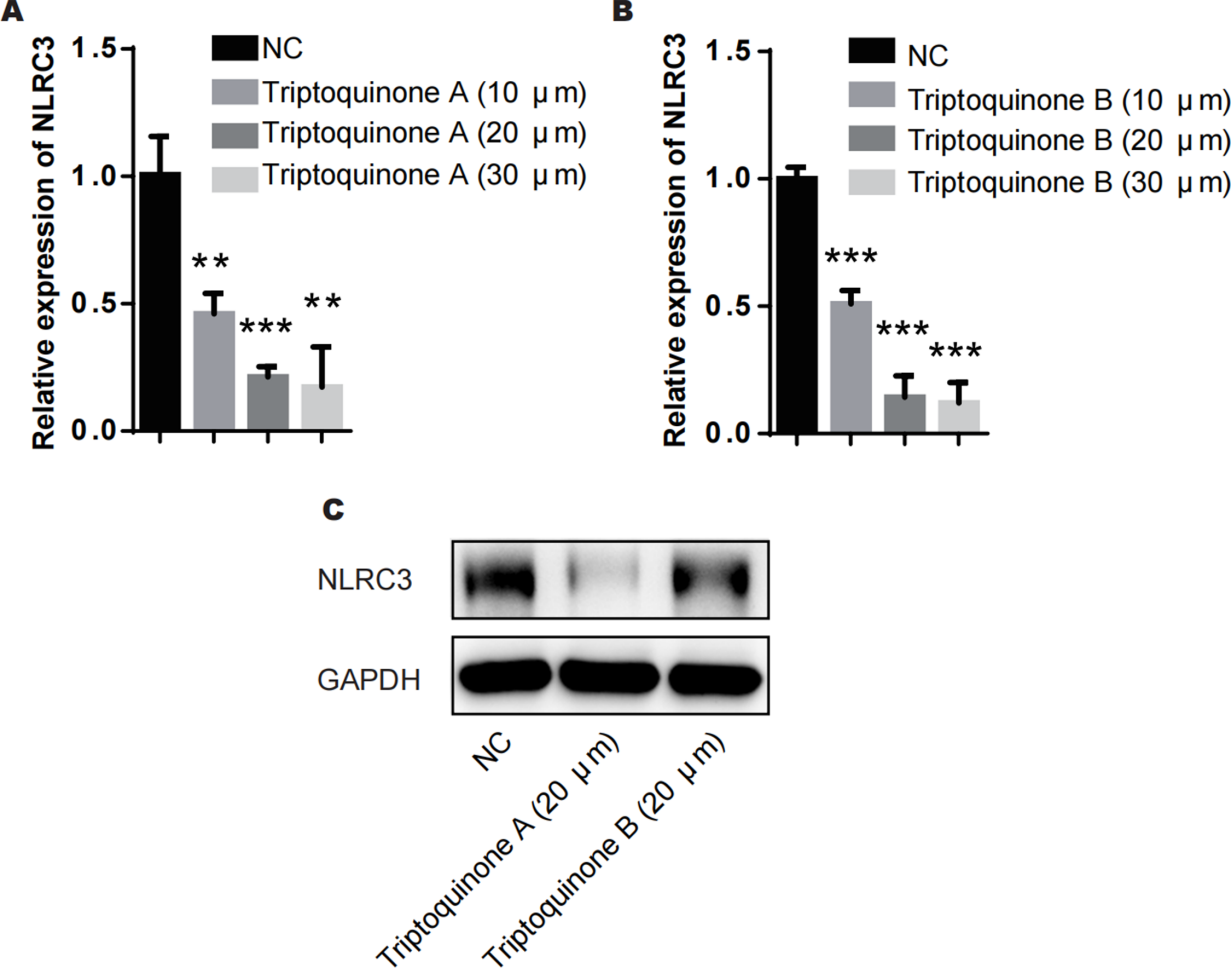
Triptoquinone A and Triptoquinone B inhibit NLRC3 expression. (A) Triptoquinone A was found to inhibit NLRC3 expression by qRT-PCR, with expression decreasing gradually as concentration increased; (B) Triptoquinone B inhibited NLRC3 expression by qRT-PCR, with expression decreasing gradually as concentration increased; (C) Triptoquinone A and Triptoquinone B inhibited NLRC3 expression by western blot assay, revealing that both compounds could suppress NLRC3 expression; (D) ELISA experiments demonstrated that in the IL-1β-induced inflammation group, the expression of pro-inflammatory cytokines TNF-α and IL-6 increased significantly but exhibited a dose-dependent decrease as Triptoquinone A and Triptoquinone B concentrations increased; (E) CCK8 cell viability assays revealed a substantial reduction in chondrocyte vitality following IL-1β induction; (F) qRT-PCR experiments indicated that cytokine IL-8 expression and cartilage-degrading enzyme MMP-13 levels significantly rose in the inflammation model group, but displayed a dose-dependent decline as Triptoquinone A and Triptoquinone B concentrations increased.

From our analysis, it is evident that there exists a relatively intimate association between triptoquinone A, triptoquinone B, and NLRC3. Despite previous research suggesting that NLRC3 may not exhibit differential expression in SLE and normal tissue (Shao et al., 2016), chondrocytes treated with triptoquinone A and triptoquinone B were scrutinized utilizing quantitative real-time PCR (qRT-PCR) and Western blot analyses (Fig. 6). Following treatment with triptoquinone A and triptoquinone B, chondrocytes displayed a downregulation of NLRC3 expression (Figs. 6A–6C). Furthermore, ELISA experiments revealed an increase in pro-inflammatory cytokines TNF- *α* and IL-6 within the IL-1 *β*-induced inflammation group; however, their levels exhibited a dose-dependent decline as triptoquinone A and triptoquinone B concentrations increased (Fig. 6D). CCK8 cell viability assays demonstrated a marked reduction in chondrocyte vitality subsequent to IL-1 *β* induction (Fig. 6E). In order to thoroughly evaluate the extent of inflammatory damage in chondrocytes, we further investigated the expression of the cytokine IL-8 as well as the cartilage-degrading enzyme MMP-13 (Fig. 6F). Our findings indicate that these markers experienced a significant upsurge within the inflammation model group; nonetheless, they also responded to triptoquinone A and triptoquinone B with a dose-dependent reduction.

### The role of NLRC3 in mediating the anti-inflammatory and anti-cartilage degradation properties of triptoquinone A and triptoquinone B

**Figure 7:**
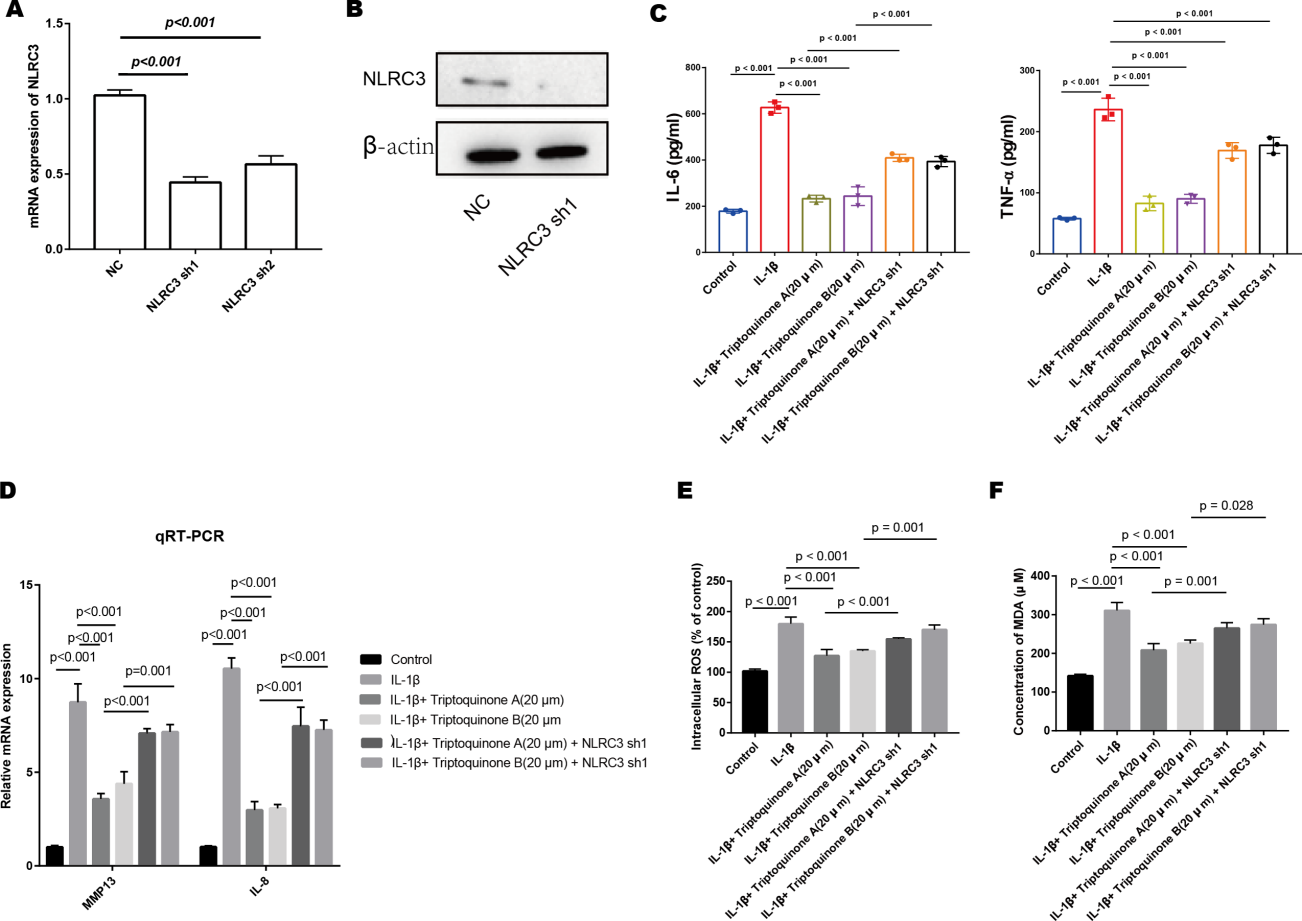
The role of NLRC3 in mediating the anti-inflammatory and anti-cartilage degradation effects of Triptoquinone A and Triptoquinone B. (A & B) Successful reduction of NLRC3 expression in chondrocytes as demonstrated by quantitative PCR (A) and Western blot (B) analyses. (C) Significant upregulation of pro-inflammatory cytokines (TNF- *α* and IL-6) in the NLRC3 silenced group compared to the control group following treatment with Triptoquinone A and Triptoquinone B. (D) Elevated expression of the cartilage-degrading enzyme MMP-13 and IL-8 in the NLRC3 silenced group compared to the control group. (E & F) Reduction of ROS (E) and MDA (F) levels following treatment with Triptoquinone A and Triptoquinone B; however, this protective effect is attenuated after silencing NLRC3.

We successfully diminished the expression of NLRC3 in chondrocytes, as demonstrated by quantitative PCR and Western blot analyses (Figs. 7A & 7B). After treatment with triptoquinone A and triptoquinone B, a comparison between the control and NLRC3 silencing groups demonstrates that both groups produce dramatically more pro-inflammatory cytokines. NLRC3 silencing also resulted in significantly higher expression levels of cartilage-degrading enzymes MMP-13 and IL-8 when compared with control cells (Figs. 7C & 7D). To further elucidate the effects of triptoquinone A and triptoquinone B on ROS and MDA levels, we evaluated them using the DCFH-DA fluorescent probe method. The experimental results showed that treatment with triptoquinone A and triptoquinone B was accompanied by a decrease in ROS and MDA levels (Figs. 7E & 7F). However, this protective effect is attenuated after silencing NLRC3. According to our findings, NLRC3 is critical for mediating the anti-inflammatory and anti-cartilage degradation properties of triptoquinone A and triptoquinone B. The silencing of NLRC3 attenuated the protective properties of triptoquinone A and triptoquinone B, suggesting that targeting NLRC3 may be a potential therapeutic approach for inflammatory and cartilage degenerative diseases.

## Discussion

Multifaceted pathogenesis and a wide variety of clinical manifestations characterize SLE, a heterogeneous autoimmune disease. Continuous advancements in the understanding of SLE pathways, biomarkers, and clinical data have led to the development of innovative drugs and therapeutic strategies for improved disease management. Tofacitinib may improve cardiometabolic status in SLE patients and prevent atherosclerosis, according to Prof. Hasni’s study (Hasni et al., 2021). The safety of fenibutinib was established, but its efficacy remains unconfirmed using the SRI-4 assessment (Isenberg et al., 2021). Contemporary pharmacological research has demonstrated that TWHF polysaccharides possess substantial anti-inflammatory properties, suggesting that their combined application could significantly mitigate the body’s inflammatory response (Wang et al., 2016). TGTs exhibited remarkable immunomodulatory capabilities, enhancing the immune function of model rats (Chen et al., 2015). This study provided evidence that TGTs may serve as a potential preventive approach against SLE arthritis, thereby presenting a new direction for SLE therapeutic interventions.

Triptoquinone is a natural compound derived from TWHF, used for treating fever, chills, edema, and carbohydrates. It also demonstrates potent cytotoxic, immunomodulatory, and anti-inflammatory properties. For instance, triptoquinone H has robust immunosuppressive capabilities. Triptoquinone A significantly inhibits the release of interleukin-1a and b from human peripheral blood mononuclear cells, while triptoquinone B is a potential treatment for RA (Cao et al., 2016). These results support our hypothesis that the active components of TGTs, triptoquinone A and triptoquinone B, may aid in treating SLE arthritis.

We found that triptoquinone A and B, the active compounds in TGTs, could potentially prevent SLE-related arthritis onset by targeting NLRC3. Our investigation involved screening the active ingredients of TGTs and identifying potential genes using a bioinformatics analysis of SLE-related diseases. The analysis process consisted of: (1) obtaining DEGs related to “SLE,” “RA,” and osteoarthritis (OA); (2) constructing PPI networks of SLE comorbidities; (3) analyzing the biological functions and KEGG pathway enrichment of shared DEGs using R and Perl software to identify potential mechanisms of co-expressed genes. These steps led to the identification of 19 potential core genes common to the diseases, such as CCL2, IL7R, TLR1, C1QB, CD163, NCF2, LTF, RGS1, KLRD1, and CD96. These genes are associated with cellular autophagy, immune regulation, and oxidative stress. Using an online Venn diagram tool, we then screened four genes related to dysregulated genes shared by TGT’s active ingredients and SLE and arthritis, including NLRC3, CD96, CCL2, and TLR1.

Existing research has identified NLRC3 as a factor involved in host immunity against pathogens such as bacteria and parasites, attenuating autoimmunity by downregulating antigen-presenting function of dendritic cells through the p38 signaling pathway (Zhang et al., 2019b; Wang et al., 2019; Vargas-Lagos et al., 2019; Hu et al., 2018; Fu et al., 2019). SLE’s pathogenesis may be influenced by NLRC3, as suggested in previous studies. Shen 2018 discussed the involvement of NLRP3 in autoimmune diseases, including SLE, and proposed it as a potential therapeutic target (Shen et al., 2018). By mediating Th1 and Th17 responses, NLRP3 is critically involved in experimental autoimmune encephalomyelitis (Gris et al., 2010). Spada 2015 discusses the role of natural killer cells in SLE pathogenesis, while Smith 2015 focuses on the role of neutrophils (Smith & Kaplan, 2015; Spada, Rojas & Barber, 2015). These findings suggest that various immune cells and pathways contribute to SLE pathogenesis, with NLRC3 being one of them. Despite the relative scarcity of research on the correlation between NLRC3 and SLE, this study discovered that triptoquinone A and triptoquinone B, the active ingredients of TGTs, can identify potential genetic genes and drugs for SLE-related arthritis prevention by binding to the target molecule NLRC3. This discovery provides a direction for future research aimed at preventing the occurrence of SLE-related arthritis. Our study showed that triptoquinone A and triptoquinone B attenuate NLRC3 expression, resulting in a dose-dependent reduction in pro-inflammatory cytokines, chondrocyte viability, and cartilage degradation markers. However, silencing NLRC3 diminished the protective effects of these compounds, inflammatory and cartilage degenerative diseases may benefit from targeting NLRC3.

Chemokine CCL2, a multifunctional factor implicated in various aspects of liver pathogenesis such as acute liver injury, chronic HBV/HCV infection, cirrhosis, and tumorigenesis (Baeck et al., 2012; Mandrekar et al., 2011), is regarded as a reliable indicator of potential signal sources in SLE (Bauer et al., 2009). CCL2 activation depends on the Jak/STAT pathway induced by interferon (Loetscher, 1998), and is one of the 12 upregulated proteins in SLE (Bauer et al., 2006). CD96 is highly expressed in acute myeloid leukemia (AML), T-cell acute lymphoblastic leukemia (T-ALL), and myelodysplastic syndromes (Meyer et al., 2009; Zhang et al., 2015) and has been proposed as a cancer stem cell marker for leukemia (Hosen et al., 2007). However, studies related to CD96 and SLE are limited. TLR1 has been reported to suppress leukemia cancer cells, and recent studies have identified the reversal of HIV-1 latency by TLR1 (Cen et al., 2019; Duan et al., 2021). TLR1 is highly expressed on the surface of peripheral blood immune cells in SLE patients (Wong et al., 2009).

Exposure to adverse factors such as oxidative stress activates signaling pathways related to inflammation and apoptosis, leading to extracellular matrix (ECM) degradation and, ultimately,the development of SLE. This study, however, has its limitations. Methodologically, it relies exclusively on bioinformatics, lacking clinical cohort validation. Additionally, the conclusions drawn from bioinformatics have not been substantiated through cellular and animal experimentation. Future research should undertake such experiments to verify the findings and design clinical cohort investigations for potential drug trials involving the active constituents. In terms of methodology, this study used pure bioinformatics analysis and molecular dynamics analysis, which lacked clinical cohort validation. Secondly, the conclusions obtained from bioinformatics also lacked cellular and animal experimental validation. The next step could be to conduct cellular and animal experiments to validate the results of bioinformatics analysis and further design clinical cohort studies for clinical trials of the active ingredients of the drug.

## Conclusion

In summary, our study demonstrates that triptoquinone A and triptoquinone B effectively alleviate SLE symptoms by targeting oxidative stress, inflammation, and chondrocyte apoptosis. NLRC3 signaling is closely linked to the mechanisms underlying these effects. It is therefore possible that triptoquinone A and triptoquinone B may be effective in treating SLE. By investigating the potential therapeutic effects of triptoquinone A and triptoquinone B on SLE, this study contributes to the growing body of knowledge surrounding SLE pathogenesis and treatment strategies. Although the study has some limitations, it offers valuable insights into the role of NLRC3 and the potential of triptoquinone A and B in addressing SLE-related arthritis. Further research should focus on validating these findings through cellular and animal experimentation and exploring clinical cohort studies to assess the viability of these active ingredients for drug trials. Ultimately, the development of novel therapeutic approaches for SLE could significantly improve the quality of life for patients suffering from this complex autoimmune disease.

##  Supplemental Information

10.7717/peerj.15395/supp-1 Supplemental Information 1 Raw plots, raw data and code

## Abbreviations

SLE: Systemic lupus erythematosus
RA: Rheumatoid arthritis
GEO: Gene expression omnibus
TGTs: Tripterysium Glycosides tablets

## Additional Information and Declarations

### Data Availability

The following information was supplied regarding data availability: The raw measurements are available in the Supplementary File.
