# Supplementary figures and images for: Triptoquinone A and B exercise a therapeutic effect in systemic lupus erythematosus by regulating NLRC3
Source: PeerJ. 2023 Jun 9;11:e15395. doi: 10.7717/peerj.15395 (PMC10259444; doi:10.7717/peerj.15395)

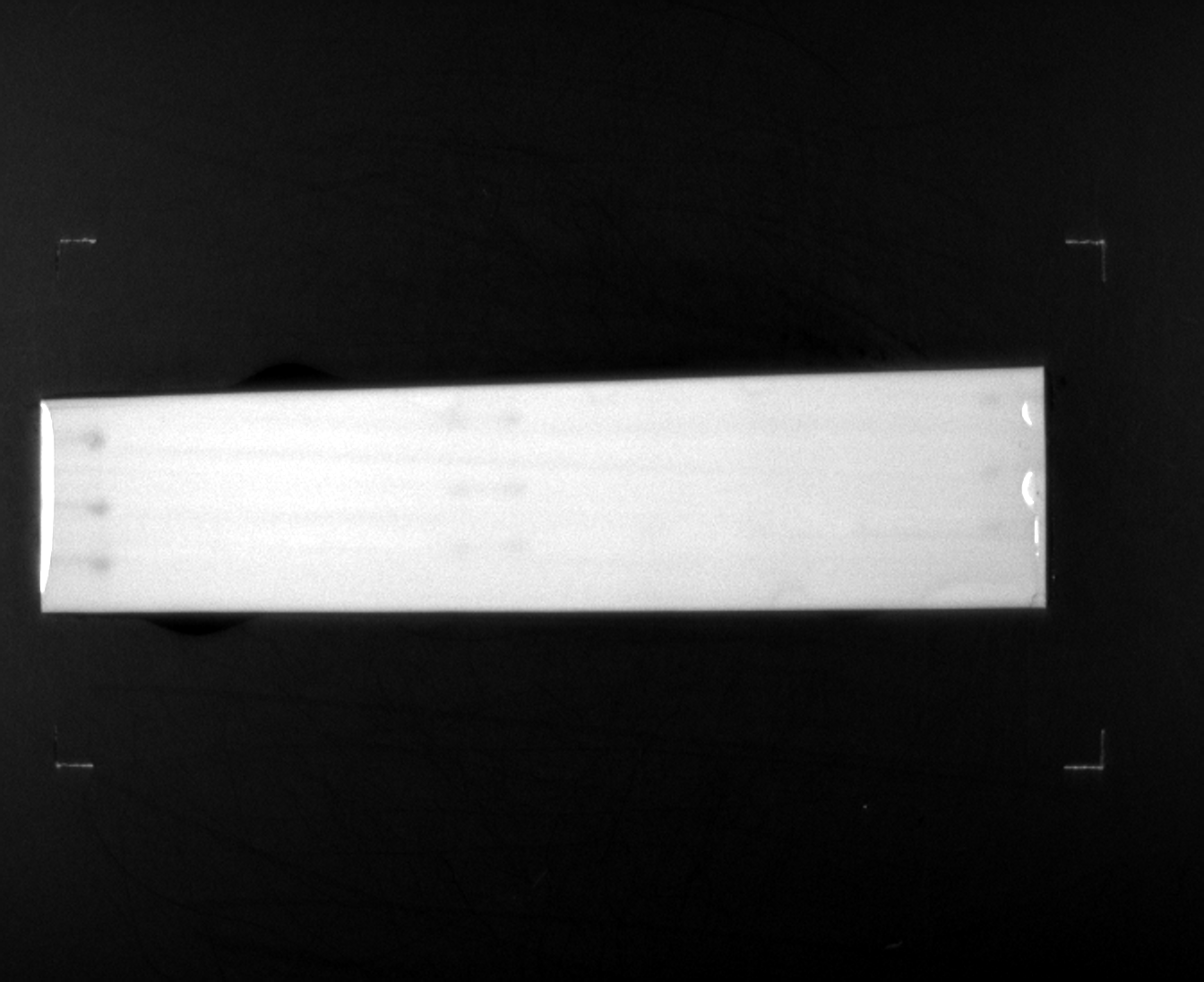

Supplement: Supplemental Information 1 [file peerj-11-15395-s001.zip › rawdata/figure6WB/GAPDH/0.Tif]

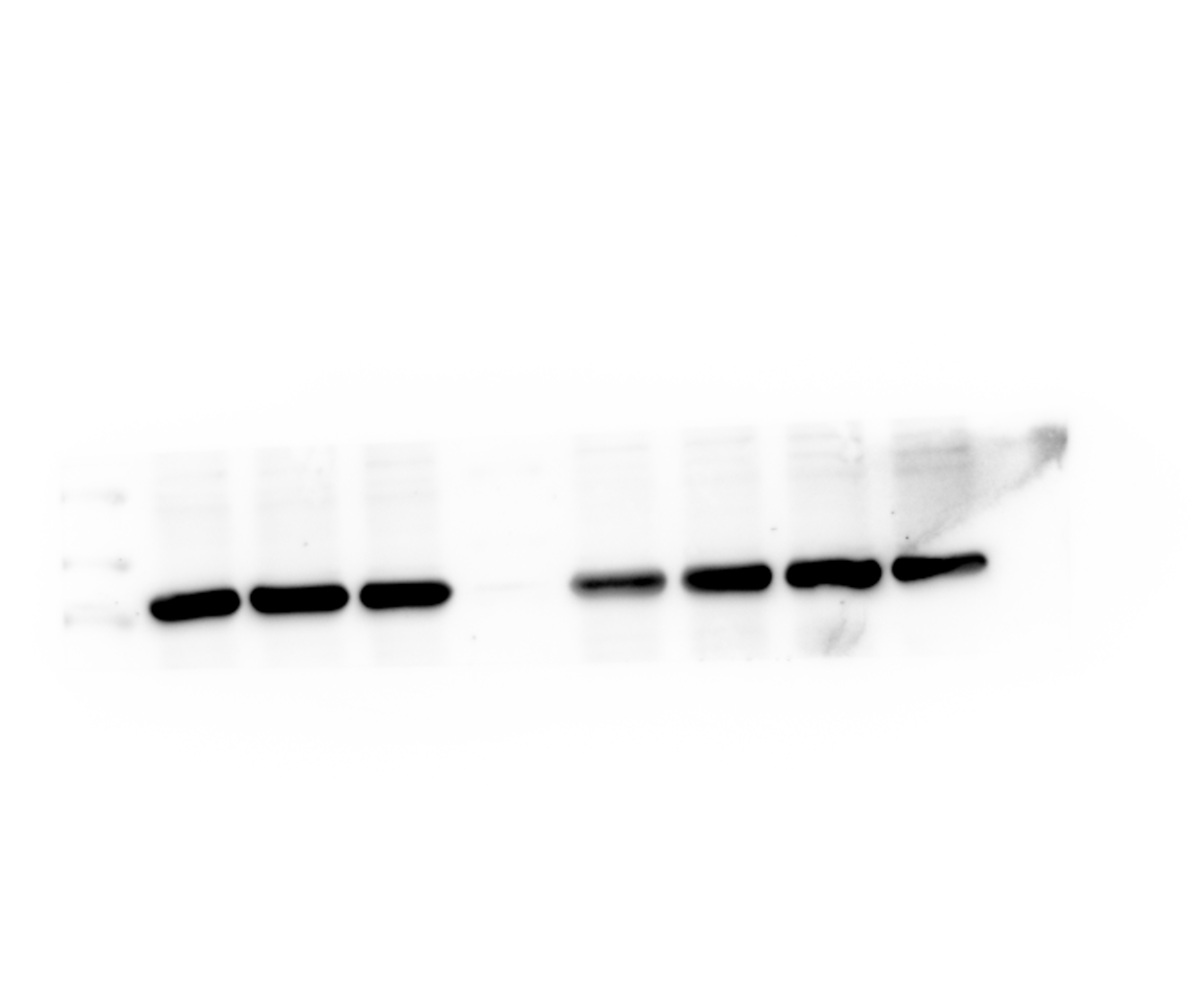

Supplement: Supplemental Information 1 [file peerj-11-15395-s001.zip › rawdata/figure6WB/GAPDH/10.Tif]

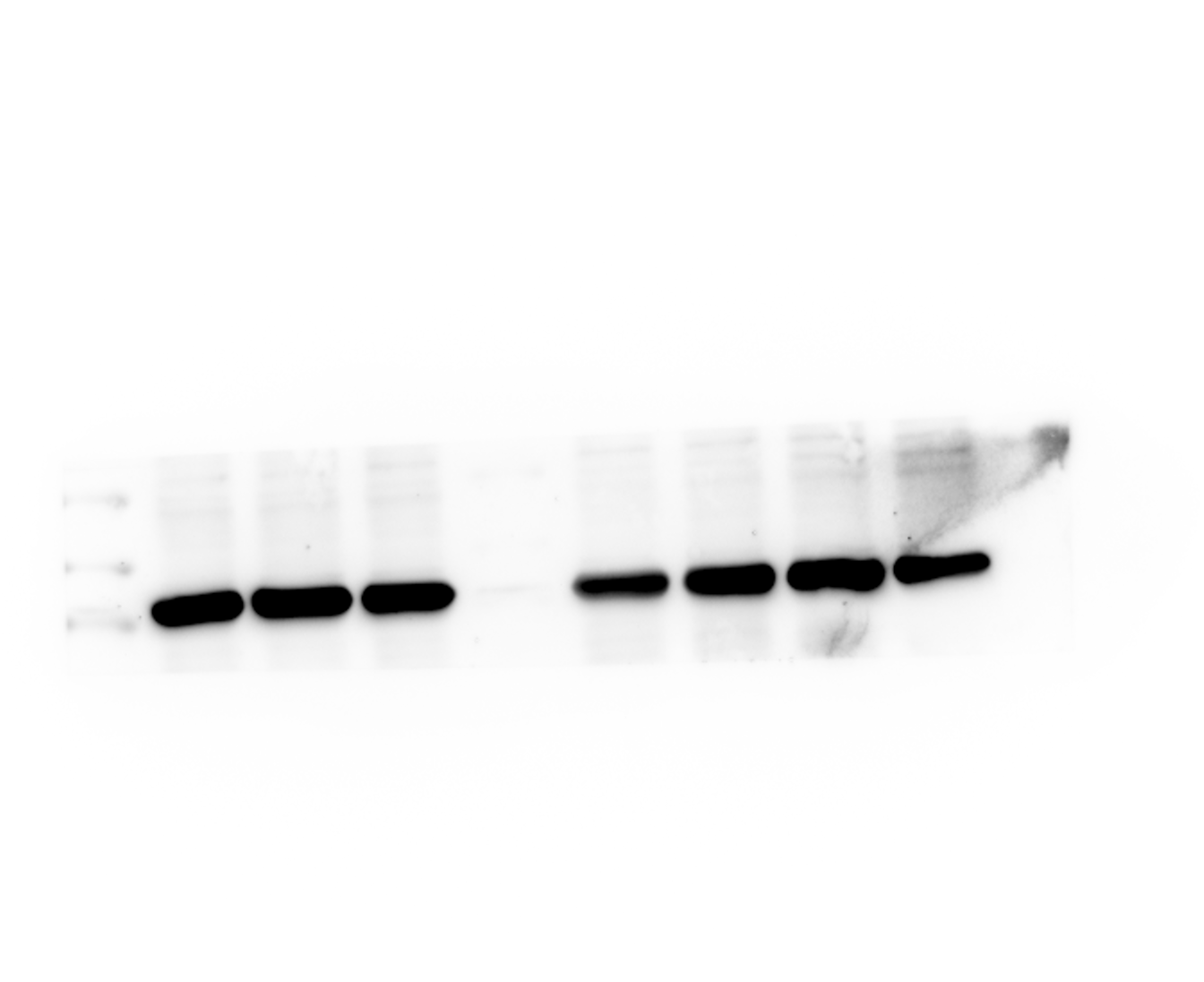

Supplement: Supplemental Information 1 [file peerj-11-15395-s001.zip › rawdata/figure6WB/GAPDH/15.Tif]

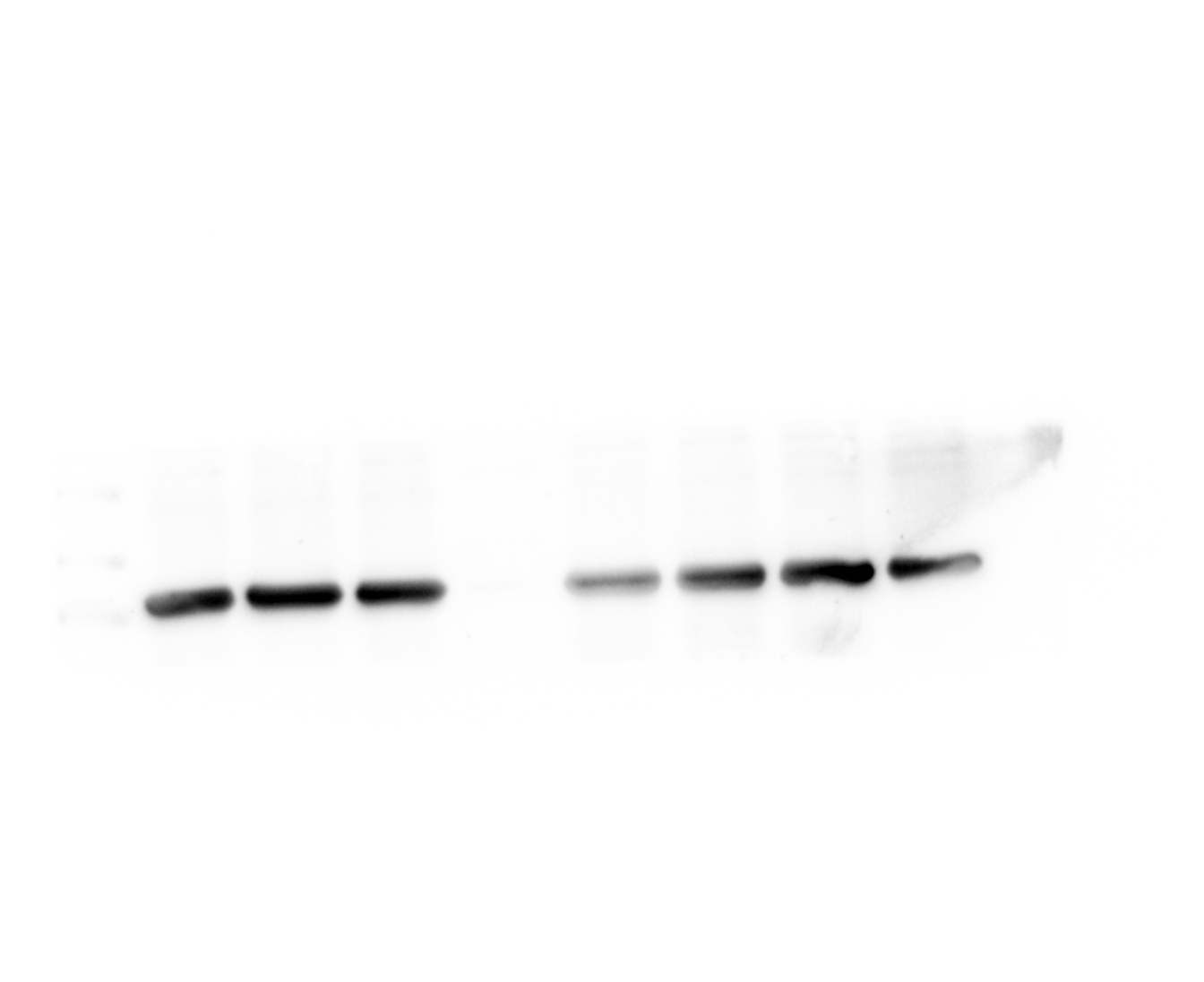

Supplement: Supplemental Information 1 [file peerj-11-15395-s001.zip › rawdata/figure6WB/GAPDH/2.2.Tif]

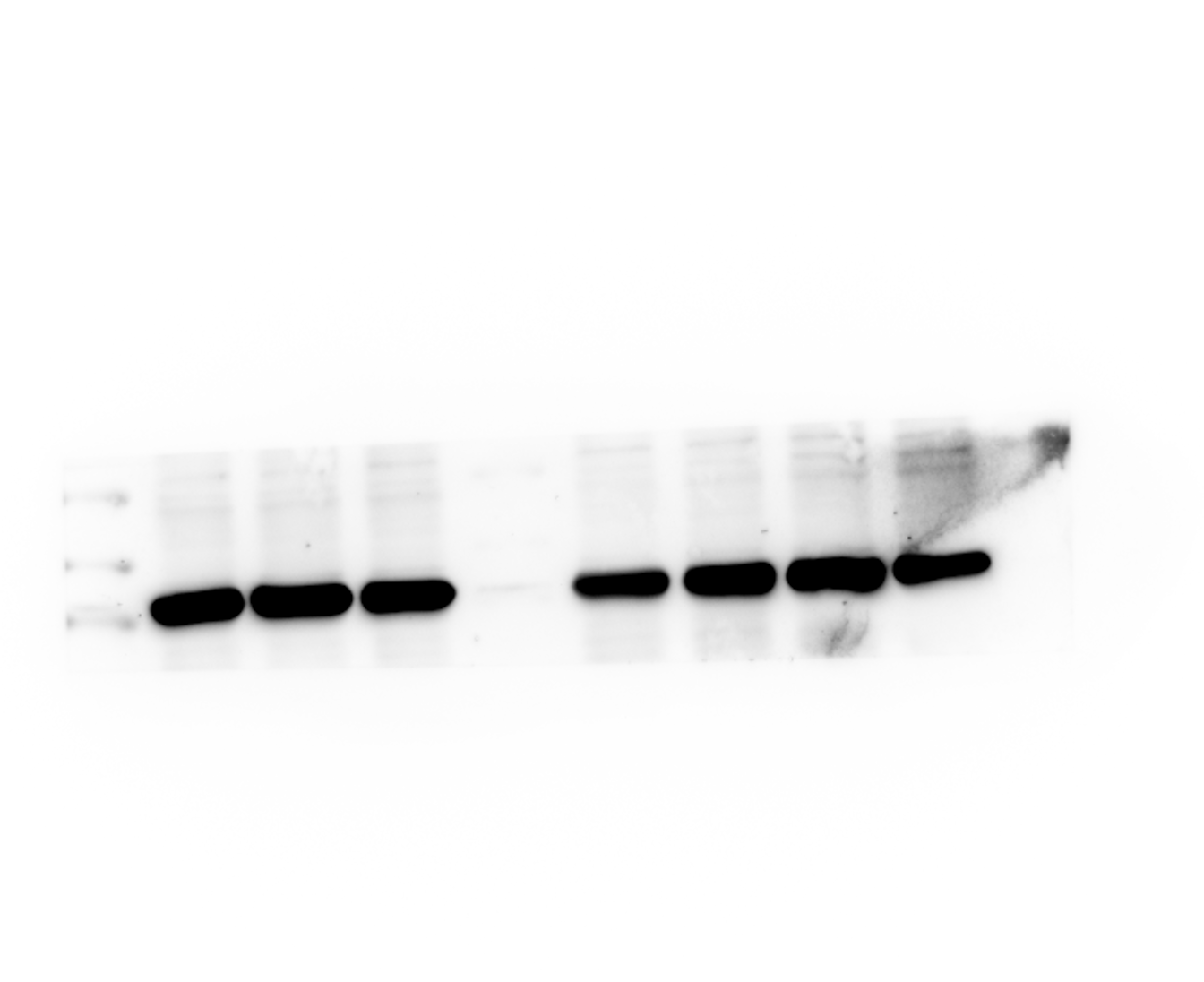

Supplement: Supplemental Information 1 [file peerj-11-15395-s001.zip › rawdata/figure6WB/GAPDH/20.Tif]

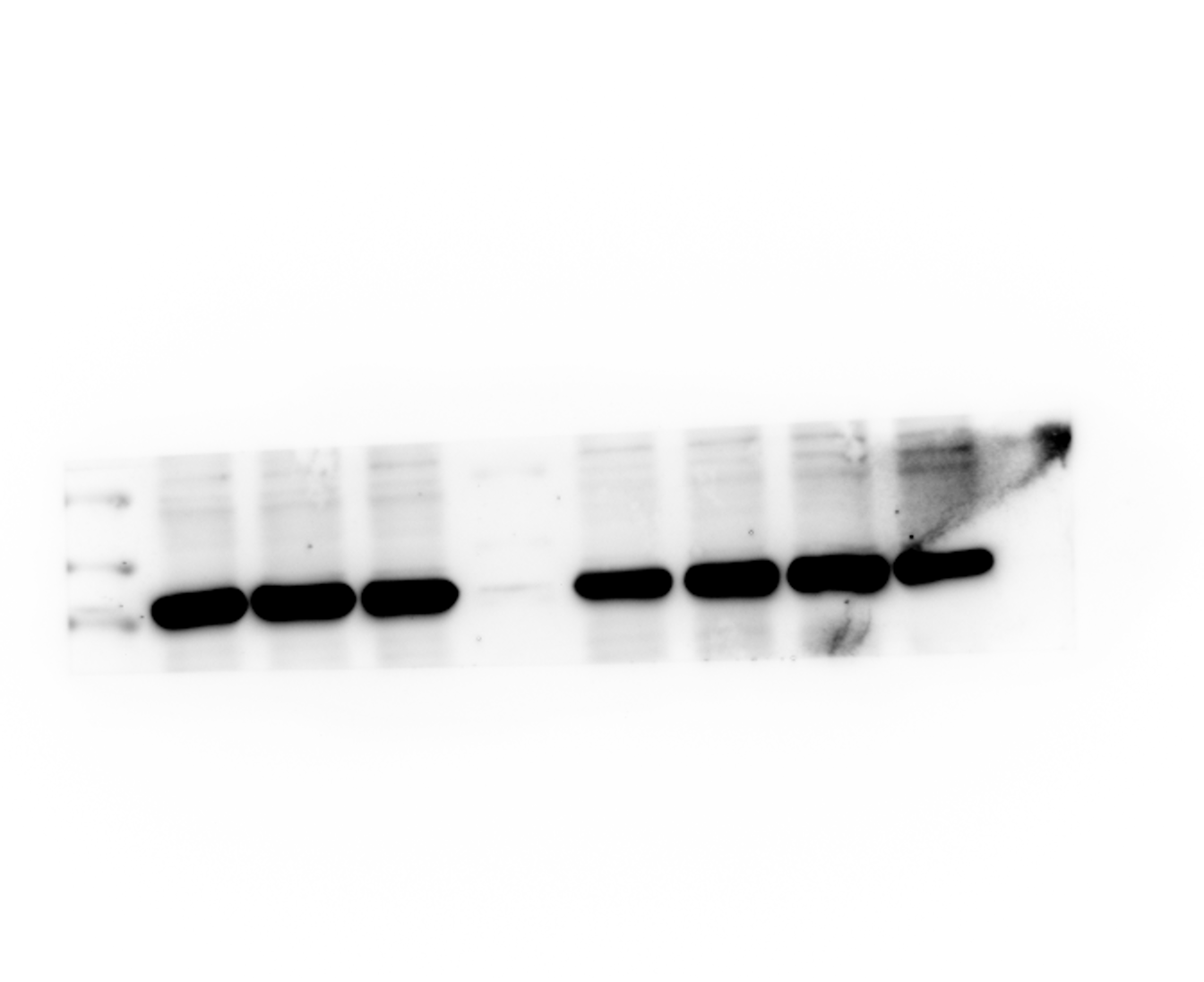

Supplement: Supplemental Information 1 [file peerj-11-15395-s001.zip › rawdata/figure6WB/GAPDH/30.Tif]

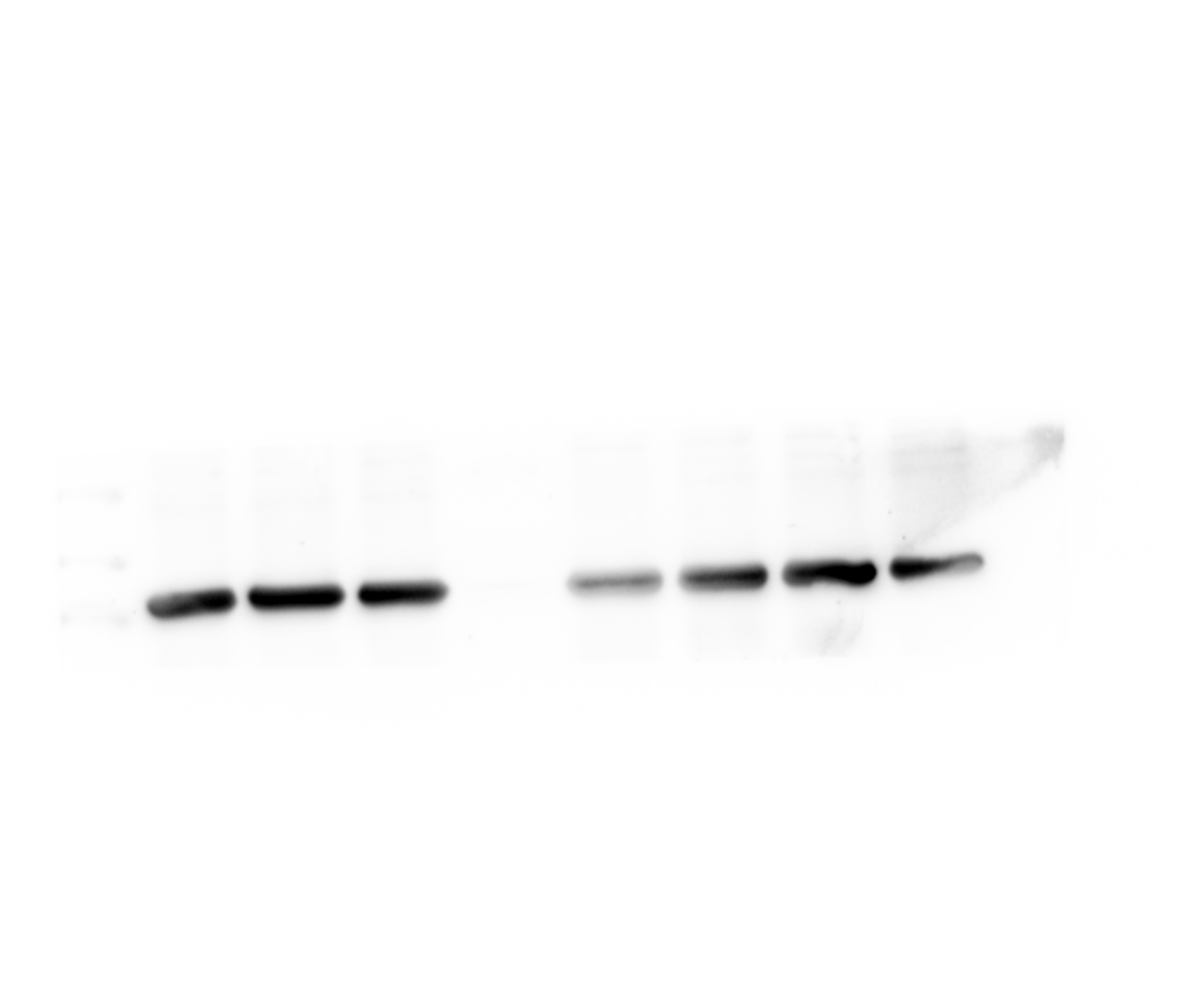

Supplement: Supplemental Information 1 [file peerj-11-15395-s001.zip › rawdata/figure6WB/GAPDH/5.Tif]

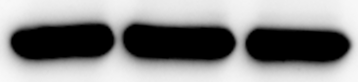

Supplement: Supplemental Information 1 [file peerj-11-15395-s001.zip › rawdata/figure6WB/GAPDH/雷公藤-GAPDH.tif]

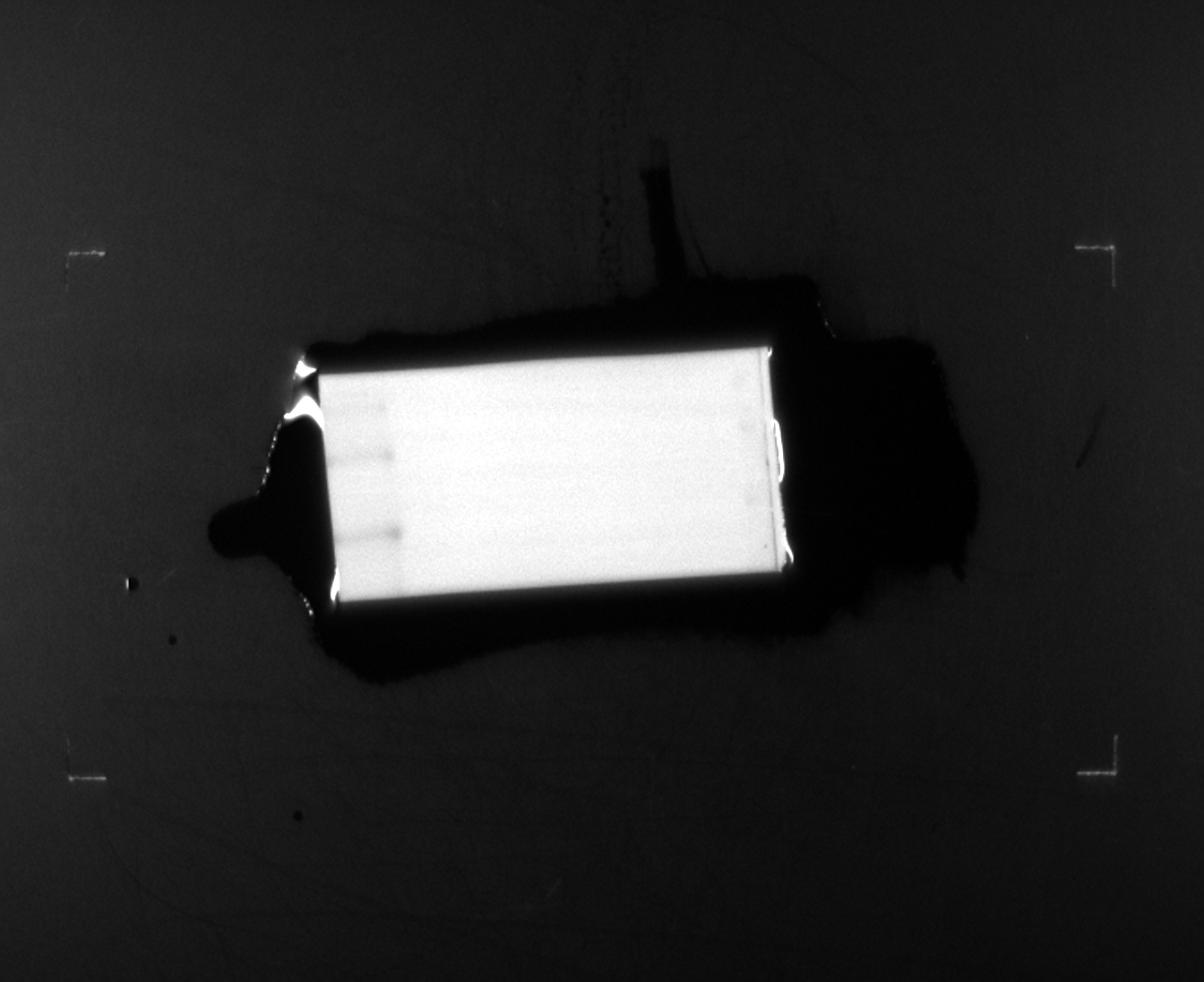

Supplement: Supplemental Information 1 [file peerj-11-15395-s001.zip › rawdata/figure6WB/NLRC-3/0.Tif]

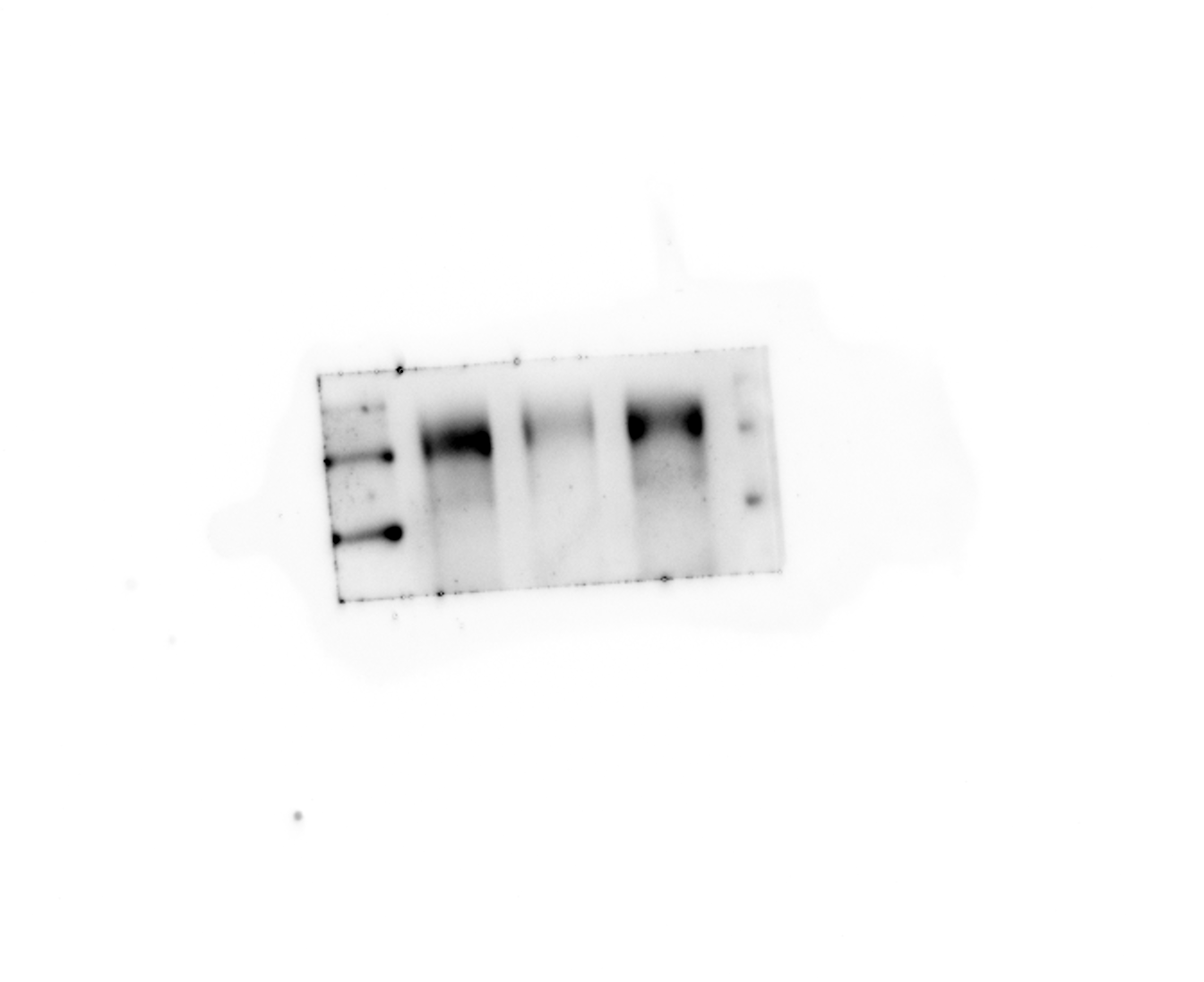

Supplement: Supplemental Information 1 [file peerj-11-15395-s001.zip › rawdata/figure6WB/NLRC-3/100.Tif]

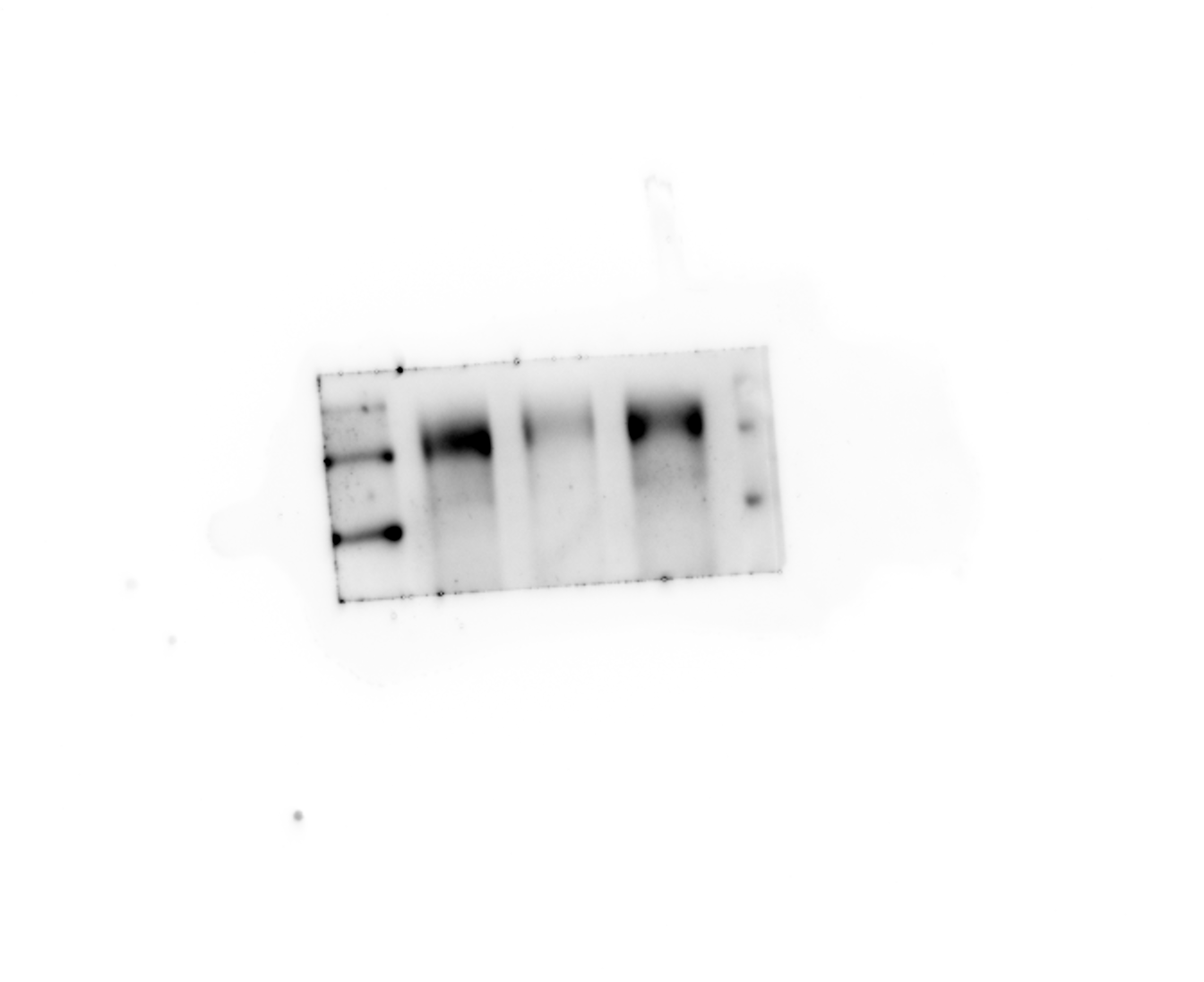

Supplement: Supplemental Information 1 [file peerj-11-15395-s001.zip › rawdata/figure6WB/NLRC-3/300.Tif]

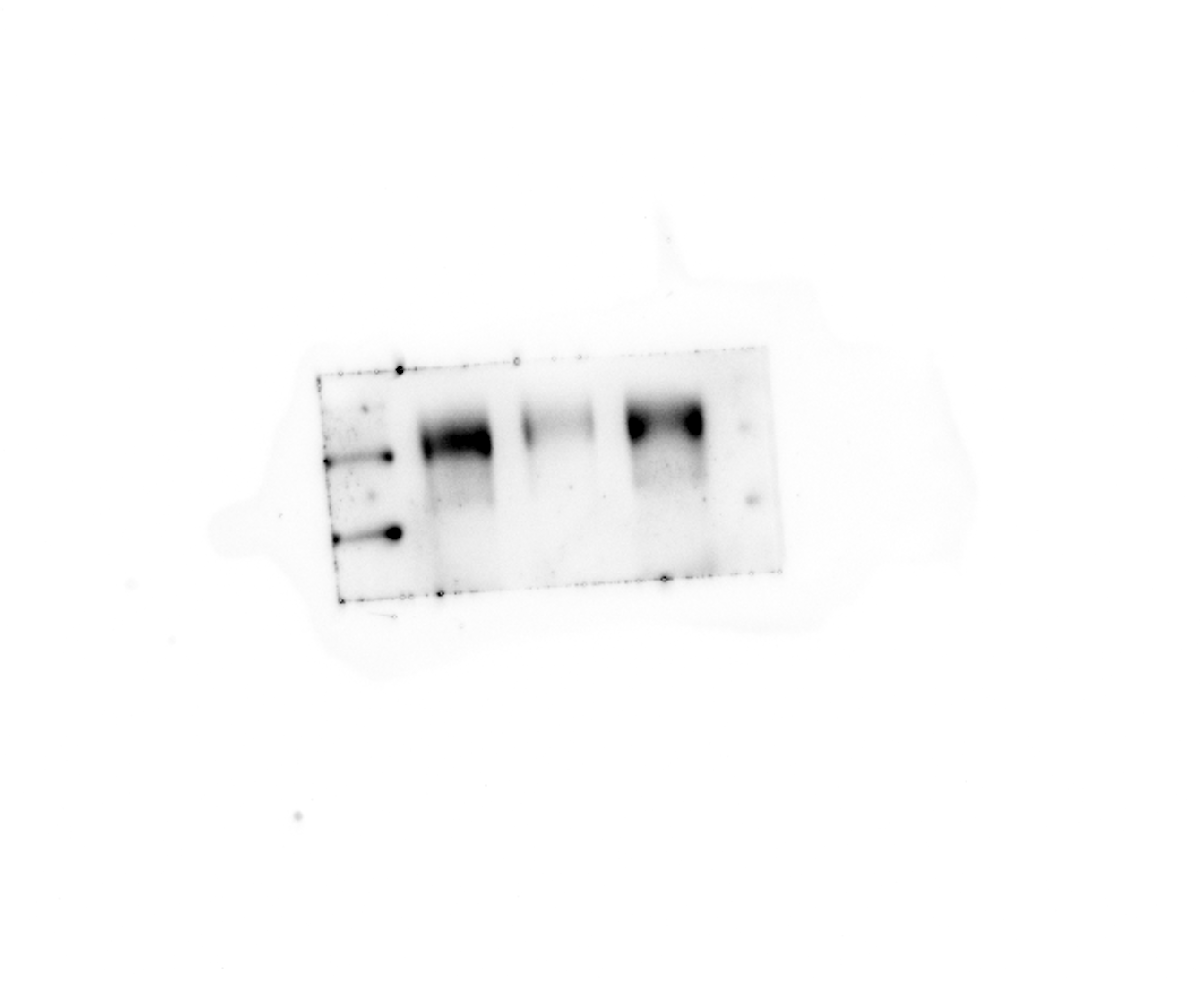

Supplement: Supplemental Information 1 [file peerj-11-15395-s001.zip › rawdata/figure6WB/NLRC-3/67.Tif]

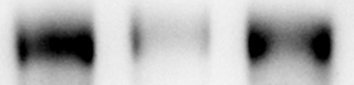

Supplement: Supplemental Information 1 [file peerj-11-15395-s001.zip › rawdata/figure6WB/NLRC-3/雷公藤-NLRC-3.tif]

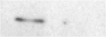

Supplement: Supplemental Information 1 [file peerj-11-15395-s001.zip › rawdata/figure8WB rawdata/figure8 NLRC3 (2).png]

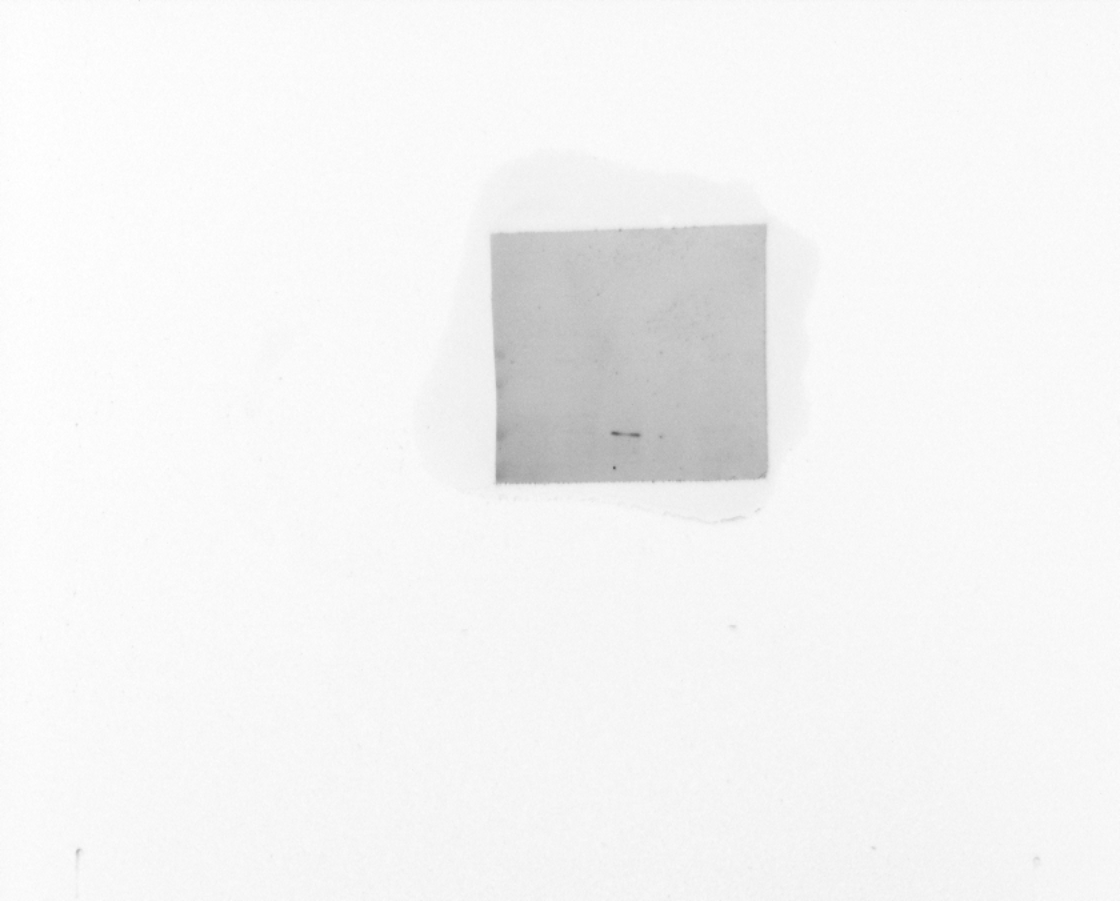

Supplement: Supplemental Information 1 [file peerj-11-15395-s001.zip › rawdata/figure8WB rawdata/figure8 NLRC3 (2).tif]

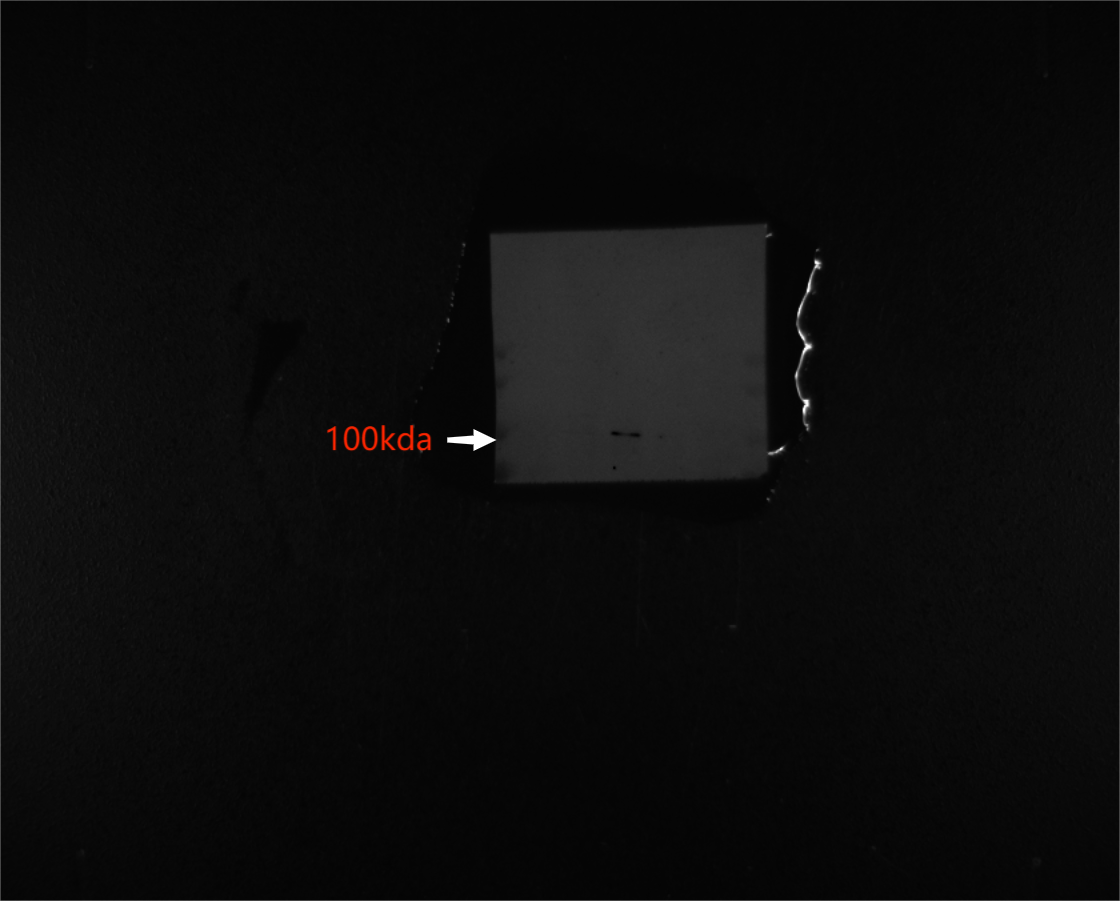

Supplement: Supplemental Information 1 [file peerj-11-15395-s001.zip › rawdata/figure8WB rawdata/figure8 NLRC3.png]

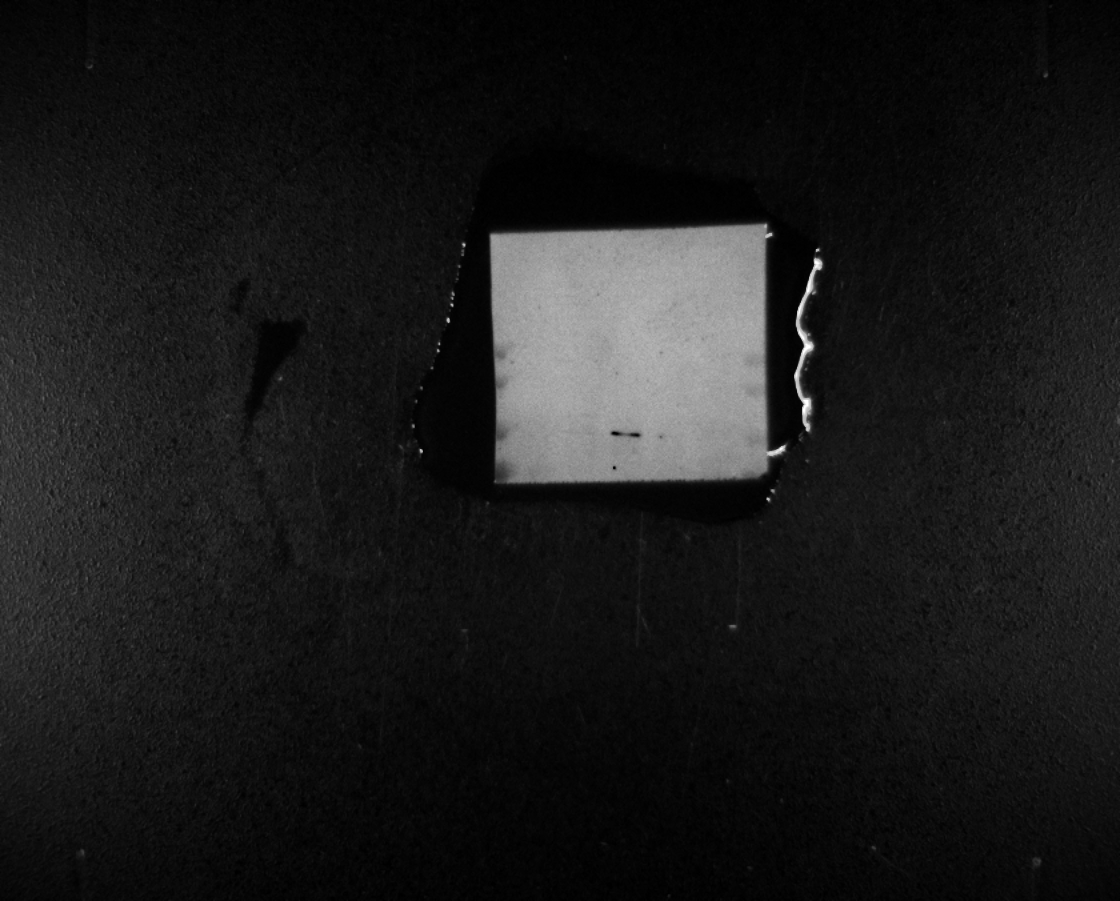

Supplement: Supplemental Information 1 [file peerj-11-15395-s001.zip › rawdata/figure8WB rawdata/figure8 NLRC3.tif]

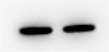

Supplement: Supplemental Information 1 [file peerj-11-15395-s001.zip › rawdata/figure8WB rawdata/figure8β-actin_58s (2).png]

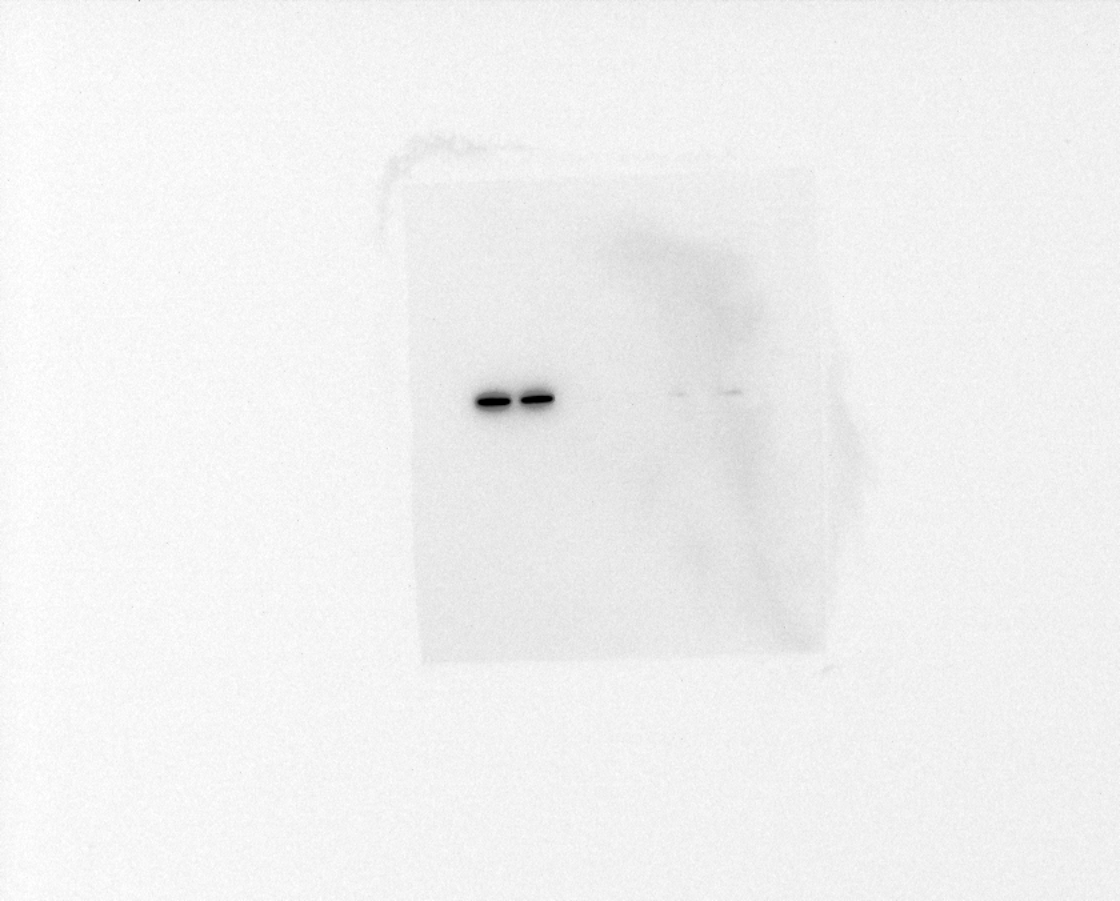

Supplement: Supplemental Information 1 [file peerj-11-15395-s001.zip › rawdata/figure8WB rawdata/figure8β-actin_58s (2).tif]

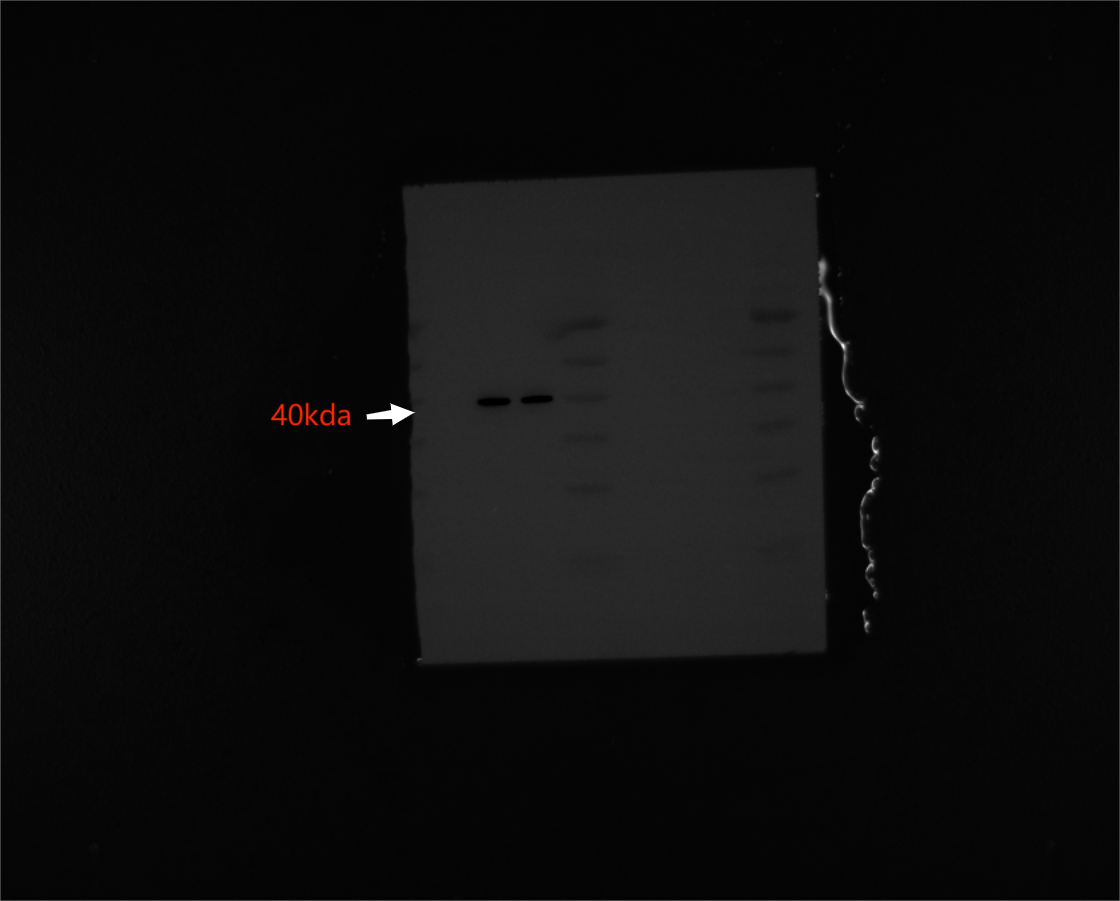

Supplement: Supplemental Information 1 [file peerj-11-15395-s001.zip › rawdata/figure8WB rawdata/figure8β-actin_58s.png]

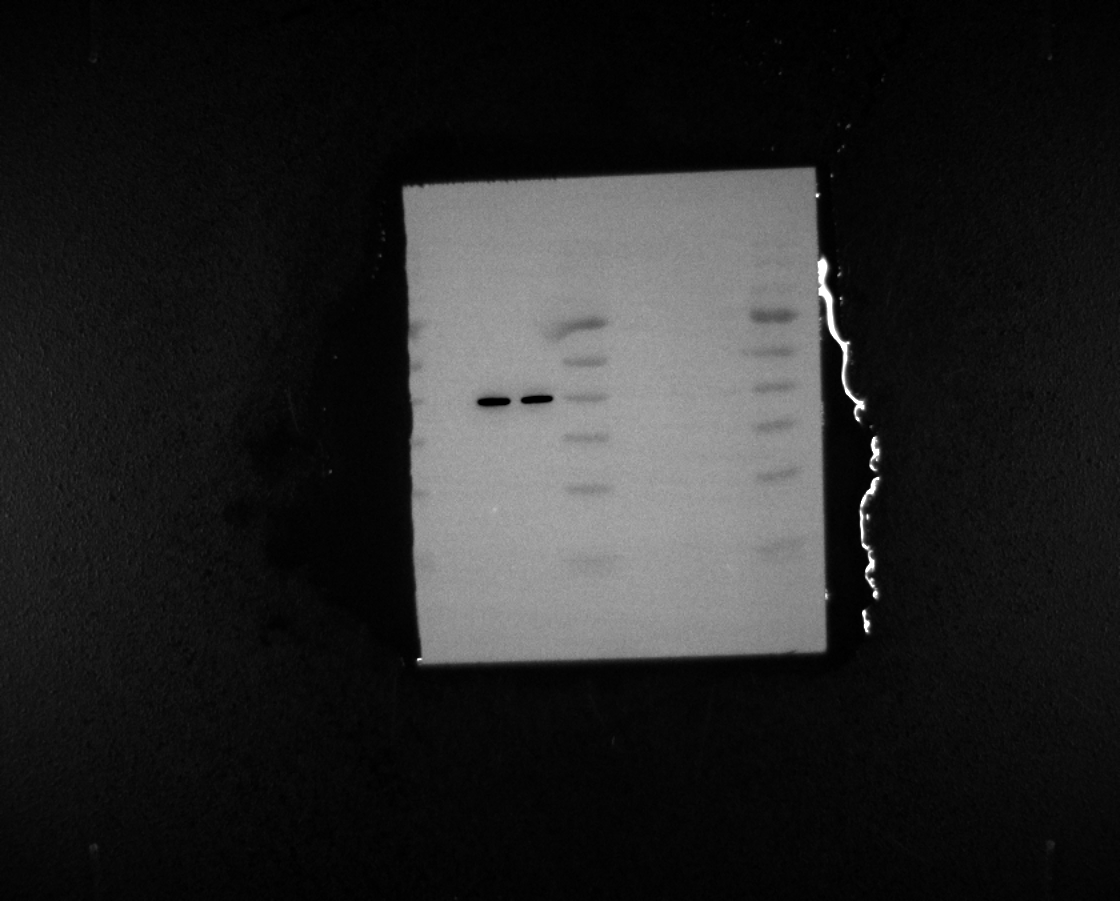

Supplement: Supplemental Information 1 [file peerj-11-15395-s001.zip › rawdata/figure8WB rawdata/figure8β-actin_58s.tif]
